# Performance of seven carbapenemase detection assays in *Pseudomonas aeruginosa* across different epidemiological settings: a multicenter cross-sectional study

**DOI:** 10.1128/jcm.00714-26

**Published:** 2026-09-17

**Authors:** Lukas Schaffarczyk, Rebekka Laufenberg, Timo J. B. van Eldijk, Niels Pfennigwerth, Sören Gatermann, Axel Hamprecht

**Affiliations:** 1 Department of Medical Microbiology and Virology, Carl von Ossietzky Universität Oldenburg 11233, Oldenburg, Germany; 2 Institute of Medical Microbiology and Virology, Klinikum Oldenburg 37099, Oldenburg, Germany; 3 SYNLAB MVZ Leverkusen GmbH 603592, Leverkusen, Germany; 4 Department of Medical Microbiology, Ruhr-University Bochum, German National Reference Center for Multidrug-resistant Gram-negative Bacteria 235552, Bochum, Germany; 5 Institute for Medical Microbiology, Immunology and Hygiene, University of Cologne, University Hospital 14309, Cologne, Germany; 6 German Center for Infection Research, Partner Site Bonn-Cologne, Cologne, Germany; Maine Medical Center Department of Medicine, Portland, Maine

**Keywords:** *Pseudomonas aeruginosa*, carbapenemase detection, phenotypic assays, carbapenem inactivation method, metallo-β-lactamase, diagnostic accuracy, CIM, mzCIM, mCIM, Carba NP

## Abstract

The detection of carbapenemases in *Pseudomonas aeruginosa* remains challenging due to a great variety of other resistance mechanisms, and most laboratories, therefore, do not test for them. This study aimed to comparatively evaluate seven phenotypic carbapenemase detection assays across three epidemiological settings. A total of 320 *P. aeruginosa* isolates from three German centers with varying carbapenemase prevalences (5.8%–51.4%), including 113 carbapenemase-producing isolates carrying VIM-2 (*n* = 58), NDM-1 (*n* = 19), and GIM-1 (*n* = 16), underwent whole-genome sequencing as reference to assess seven phenotypic carbapenemase-detection tests: modified- and modified-zinc-supplemented carbapenem inactivation method (mCIM and mzCIM), simplified carbapenem inactivation method (sCIM), Carba NP, imipenem–cloxacillin test (IC-4000), and two imipenem–EDTA disk assays. Of all confirmation assays, mzCIM and sCIM showed the best overall performance for carbapenemase detection (sensitivity/specificity: 100%/94.2% and 99.1%/92.8%), followed by mCIM (93.8%/96.1%). Carba NP achieved the highest specificity (99.0%), but the lowest sensitivity (85.8%). EDTA-based assays and IC-4000 were highly sensitive (96.5%–100%) but less specific (79.7%–87.0%). Negative predictive values were consistently high (≥98%–100%) across all assays and prevalence settings, whereas positive predictive values varied (72.5%–98.0%). Both mzCIM and sCIM exhibited robust performance for carbapenemase detection in *P. aeruginosa*, representing the most suitable approach across diverse epidemiological settings. Their high negative predictive values indicate that these assays are particularly effective for ruling out carbapenemase production. Furthermore, both assays are cost-effective, simple to perform, and can be readily implemented in any routine microbiology laboratory.

**IMPORTANCE:**

This study provides comparative diagnostic accuracy data for seven phenotypic carbapenemase detection assays in *Pseudomonas aeruginosa* (PA) across different prevalence settings. Modified-zinc-supplemented carbapenem inactivation method (mzCIM) and simplified carbapenem inactivation method (sCIM) are the most robust screening tools and show that local carbapenemase-producing *P. aeruginosa* (CP-PA) prevalence substantially influences the utility of all evaluated assays.

## INTRODUCTION

*Pseudomonas aeruginosa* (PA) is a gram-negative, flagellated, oxidase-positive bacterium belonging to the family Pseudomonadaceae. It is an opportunistic pathogen commonly associated with moist environments and a major cause of nosocomial infections like pneumonia, wound infections, bloodstream infections, and urinary tract infections.

The global spread of multidrug-resistant *P. aeruginosa* strains, particularly carbapenem-resistant isolates, represents a major public health concern, reflected in their classification as high-priority pathogens in the WHO Global Priority List of Antibiotic-Resistant Bacteria (1). Carbapenem resistance in PA arises through multiple mechanisms, including porin loss (e.g., OprD), efflux pump overexpression (e.g., MexAB-OprM), production of AmpC β-lactamases and ESBLs, and acquisition of carbapenemases, with a recent increase in carbapenemase-producing isolates predominantly driven by Ambler class B metallo-β-lactamases, such as VIM-2 and NDM-1 (2–9). According to the 2024 data from the German National Reference Center for Multidrug-resistant gram-negative Bacteria, carbapenemases were detected in 29.8% of 1,957 submitted *P. aeruginosa* isolates, with VIM-2 being the most frequent (16.7%). Submission criteria included resistance to imipenem, meropenem, and ceftazidime. Consequently, in approximately 70% of submitted isolates, carbapenem resistance was likely attributable to alternative resistance mechanisms rather than carbapenemase production (9).

Because of those multiple resistance mechanisms, the detection of carbapenemases is challenging, and many laboratories therefore do not test for them. Analogous to Enterobacterales, the Clinical and Laboratory Standards Institute (CLSI) recommends the modified carbapenem inactivation method (mCIM) and the Carba NP assay as phenotypic tests for the detection of carbapenemase activity in PA (10). In contrast, the European Committee on Antimicrobial Susceptibility Testing (EUCAST) does not currently endorse a specific phenotypic method, emphasizing that most disk-based assays lack robust multicenter validation and that even colorimetric tests show insufficient specificity (11). This discrepancy underscores the need for systematic comparative evaluations of phenotypic carbapenemase detection methods across different epidemiological contexts.

This study aimed to comparatively evaluate seven phenotypic carbapenemase detection methods to identify a practical and reliable assay suitable for routine diagnostics. Furthermore, the inclusion of three isolate collections with different carbapenemase prevalences enabled an exploratory assessment of the resulting positive and negative predictive values of the assays across distinct epidemiological settings.

## MATERIALS AND METHODS

### Antimicrobial susceptibility testing

Antimicrobial susceptibility testing was performed using disk diffusion testing and interpreted according to the clinical breakpoints of the EUCAST version 16.0 (valid from 01-01-2026) (12). Susceptibility testing included 12 antibiotics: piperacillin, piperacillin-tazobactam, ceftazidime, ceftazidime-avibactam, ceftolozane-tazobactam, cefepime, cefiderocol, aztreonam, imipenem, meropenem (Liofilchem, Roseto degli Abruzzi, Italy), as well as ciprofloxacin and amikacin (Oxoid, Basingstoke, UK).

### Bacterial isolates

All 320 isolates underwent whole-genome sequencing to determine the presence or absence of acquired carbapenemase genes. Isolates originated from three distinct sources. The largest group consisted of 177 isolates from the German National Reference Center for Multidrug-resistant gram-negative bacteria (NRC, high-prevalence setting), deliberately assembled as a balanced reference panel of carbapenemase-producing (*n* = 91) and carbapenemase-negative (*n* = 86) isolates. A second selection of 74 isolates was contributed by MVZ SYNLAB Leverkusen, Germany (Center 1, intermediate-prevalence setting), included based on non-susceptibility to imipenem, meropenem, and ceftazidime, in accordance with the carbapenemase screening criteria proposed by the NRC: imipenem resistance (MIC > 4 mg/L), meropenem resistance (MIC > 8 mg/L), and ceftazidime resistance (MIC > 8 mg/L). Isolates resistant to ceftolozane-tazobactam (*n* = 31) and/or ceftazidime-avibactam (*n* = 24) were additionally included. Eighteen isolates carried carbapenemase-encoding genes, and 56 were carbapenemase-negative (13).

The third group comprised 69 isolates from the Institute of Medical Microbiology and Virology, Oldenburg, Germany (Center 2, low-prevalence setting), selected on the same criteria. Of these isolates, 8/69 were ceftazidime-avibactam resistant, and 7/69 were ceftolozane-tazobactam resistant. Four isolates carried carbapenemase genes, and 65 were carbapenemase-negative.

The three groups differed substantially in their proportion of carbapenemase-producing isolates (NRC: 51.4%; Center 1: 24.3%; Center 2: 5.8%). While primary analyses were conducted on the combined overall cohort, these differences enabled exploratory, center-stratified analyses of predictive values across cohorts with varying carbapenemase prevalences.

Raw sequencing data have been deposited in the Sequence Read Archive of the National Center for Biotechnology Information under BioProject accession number PRJNA1459623. Individual accession numbers are provided in Table S1. All tests were performed on the same day and read by a person who was blinded to the molecular characterization results of the isolates.

### Whole-genome sequencing

Genomic DNA was extracted from overnight cultures using Qiagen DNeasy UltraClean Kit and subjected to whole-genome sequencing using paired-end Illumina sequencing (2 × 150 bp) (Illumina, San Diego, USA) as previously described (14–16). After sequencing, the reads were analyzed using an automated assembly and analysis pipeline (17). In this pipeline, the reads were first filtered using FASTP (18), and an automated report was compiled using FastQC (19). Next, short-read-only genome assembly was performed using Unicycler (20), and the resulting assembly was analyzed using assembly-stats (21). Next, BLAST+ (22) was used to check the identity of a subset of the assembled contigs using the “*nt*,” “*Refseq Prokaryotic*,” and “*16S ribosomal*” databases. Subsequently, the assembly was analyzed using ResFinder v4.7.2, using default thresholds of 90% nucleotide identity and 60% gene coverage (23). Finally, the results of the entire pipeline (sequencing analysis, assembly, Blast+ identification, and ResFinder results) were compiled into a single report using rmarkdown (24).

### Phenotypic methods

### Modified carbapenem inactivation method

mCIM was performed as specified by the Clinical and Laboratory Standards Institute (10). Briefly, a 10-μL loop full of bacteria was inoculated in 2 mL Brain Heart Infusion (BHI; BD, Heidelberg, Germany) and vortexed for 15 s. A 10 µg meropenem disk (Oxoid, Wesel, Germany) was added to the suspension and incubated for 4 h at 35°C ± 1°C in ambient air, before the disk was placed on a Mueller-Hinton agar plate (MHA; Oxoid, Wesel, Germany), previously inoculated with a susceptible *Escherichia coli* indicator strain (ATCC 25922; 0.5 McFarland). The inhibition zone was measured after an 18 h incubation at 35°C ± 1°C in ambient air.

Zone diameters of 6–15 mm or the presence of pinpoint colonies within a 16–18 mm zone were regarded as a positive result. Zone diameters of ≥19 mm were interpreted as negative, while 16–18 mm or a diameter of ≥19 mm with the presence of pinpoint colonies within the zone were considered indeterminate.

### Modified zinc-supplemented carbapenem inactivation method

The mzCIM was carried out as previously described (25, 26). Briefly, two full 10 µL inoculation loops of bacterial colonies were suspended in 400 µL Brain Heart Infusion supplemented with ZnSO_4_ (1.5 mM final concentration). A 10 µg meropenem disk was added to the suspension and incubated for 4 h at 35°C ± 1°C, before the disk was placed on a Mueller-Hinton agar plate, previously inoculated with a susceptible *E. coli* indicator strain (ATCC 25922; 0.5 McFarland). The inhibition zone was measured after an 18 h incubation at 35°C ± 1°C. Thresholds were determined by receiver operating characteristic (ROC) analysis (see below). Satellite colonies in the inhibition zone were considered positive.

### Simplified carbapenem inactivation method

The simplified carbapenem inactivation method (sCIM) test was performed as described by Jing et al. (27). Briefly, imipenem disks were impregnated with the isolate to be tested. The disk was then placed upside down on an MHA plate previously inoculated with *E. coli* ATCC 25922. Plates were incubated aerobically at 35°C for 18 h. Inhibition zones of 6–20 mm or colonies within a ≤22 mm zone were considered positive, an inhibition zone ≥26 mm as negative, and a zone of inhibition of 23–25 mm as indeterminate (7).

### Carba NP

Carbapenemase production was assessed using the Carba NP test according to CLSI guidelines (10). Tubes with lysed bacterial isolates and pH indicator were incubated at 35°C–37°C and inspected visually for color change after 30 min and up to 2 h. Reading was performed as suggested by CLSI.

### Cloxacillin disk test (I-C4000) and EDTA disk screen (EDTA-750 and EDTA-930)

For both tests, bacterial suspensions of test strains were adjusted to a 0.5 McFarland standard and inoculated onto Mueller-Hinton agar plates. Imipenem disks (10 µg) without supplement or supplemented with either cloxacillin or EDTA were placed on the agar. Plates were incubated for 18 h at 35°C ± 2°C, and results were interpreted by comparing inhibition zones of disks with and without supplement.

The cloxacillin combined disk test (IC4000) was performed as described by Fournier et al. (28). Ten microliters of cloxacillin stock solution (400 mg/mL; Sigma-Aldrich, St. Louis, USA) were added to one of the imipenem disks. An increase of <6 mm around the imipenem + cloxacillin disk compared with the imipenem-only disk was interpreted as carbapenemase production, whereas an increase of ≥6 mm indicated AmpC overexpression as the likely cause of resistance.

For the EDTA disk screen, imipenem disks were supplemented either with 930 µg EDTA per disk (5 µL of a 0.5 M EDTA solution) as described by Pitout et al. (29) or with 750 µg EDTA per disk following the method of Yong et al. (30).

An increase in zone diameter of ≥7 mm in the presence of EDTA was considered indicative of metallo-β-lactamase (MBL) production.

### Statistical analysis and test performance evaluation

Diagnostic accuracy for the detection or exclusion of carbapenemase production was assessed on the combined overall cohort (*n* = 320) as the primary analysis. Test performance was quantified using sensitivity, specificity, positive predictive value (PPV), negative predictive value (NPV), and the Youden index (J = sensitivity + specificity − 1). Wilson score 95% confidence intervals were calculated for sensitivity, specificity, PPV, and NPV. Stratified analyses by center were performed as pre-specified exploratory analyses to assess the potential influence of differing carbapenemase-producing *P. aeruginosa* (CP-PA) prevalences on test performance. Given the limited number of carbapenemase-positive isolates in Center 1 (*n* = 18) and Center 2 (*n* = 4), these results are reported descriptively and should be interpreted with caution.

Pairwise comparisons of sensitivity and specificity between all seven assays were performed using McNemar’s test with continuity correction on the overall cohort only, yielding 21 test pairs per metric. *P* values were adjusted for multiple comparisons using the Benjamini-Hochberg false discovery rate (FDR) procedure, applied independently for sensitivity and specificity comparisons. An FDR-adjusted *P* value of <0.05 was considered statistically significant.

As the mzCIM test has not yet been systematically evaluated for *P. aeruginosa*, the optimal cut-off thresholds for distinguishing CP-PA from non-carbapenemase-producing isolates (nCP-PA) were unclear prior to this study. To determine the optimal cut-off for the mzCIM test, an ROC analysis was performed. The Youden index was used to identify the threshold that maximizes the sum of sensitivity and specificity.

An additional exploratory ROC analysis examined the imipenem inhibition zone diameter as a predictor of false-positive IC-4000 results among carbapenemase-negative isolates (*n* = 207); discriminatory ability was quantified using the area under the curve (AUC) with 95% confidence intervals, and the optimal zone diameter cut-off was determined by the Youden criterion.

To explore determinants of false-positive EDTA results, the absolute imipenem inhibition zone diameter (Oxoid disk) was compared between false-positive (zone difference ≥7 mm, WGS-negative) and true-negative isolates using the Wilcoxon rank-sum test and ROC analysis; the AUC was calculated with 95% confidence intervals by DeLong’s method.

Indeterminate test results were counted as negative in all primary analyses to reflect a real-world diagnostic setting (Table 2).

All statistical analyses were performed using R (version 4.3.1). Data processing and analysis were conducted using the dplyr (v1.2.1), stringr (v1.6.0), and readxl (v1.4.5) packages. Additional analyses, including ROC analysis and data visualization, were performed using the pROC (v1.19.0.1) and ggplot2 (v4.0.2) packages.

## RESULTS

### Epidemiology

A total of 320 *P. aeruginosa* isolates were included in the analysis, comprising 113 carbapenemase-positive (CP-PA) and 207 carbapenemase-negative (nCP-PA) isolates. Carbapenemase type distribution is summarized in Table 1. Among the 113 CP-PA isolates, MBLs predominated across all three cohorts. VIM-2 was the most frequent enzyme overall (*n* = 58), identified in all three groups (NRC: *n* = 46, Center 1: *n* = 8, Center 2: *n* = 4). NDM-1 was the second most common enzyme (*n* = 19; NRC: *n* = 14, Center 1: *n* = 5, with one isolate co-harboring GES-5), followed by GIM-1 (*n* = 16; NRC: *n* = 13, Center 1: *n* = 3). Three isolates carried two carbapenemase genes simultaneously: IMP-1 and DIM-1 (NRC), GES-5 and NDM-1 (NRC), and NDM-1 and VIM-4 (Center 1). With the exception of KPC-2 (*n* = 1) and GES-5 (*n* = 1), all CP-PA carried MBL-encoding genes (Table 1).

**TABLE 1:** Distribution of carbapenemase genes among *P. aeruginosa* isolates from the participating centers^*a*^

Carbapenemase gene	NRC	Center 1	Center 2	Total carbapenemases
VIM-2	46	8	4	58
NDM-1	14	5	0	19
GIM-1	13	3	0	16
IMP-1	6	0	0	6
VIM-1	3	1	0	4
DIM-1	2	0	0	2
FIM-1	1	1	0	2
IMP-7	2	0	0	2
VIM-4	1	1	0	2
GES-5	1	0	0	1
IMP-13	1	0	0	1
IMP-22	1	0	0	1
IMP-94	1	0	0	1
КРС−2	1	0	0	1
Total isolates with carbapenemase gene(s)	91	18	4	113
Total number of carbapenemase genes	93	19	4	116
Isolates without carbapenemases	86	56	65	207

^*a*^ The table summarizes the frequency of carbapenemase genes detected among *P. aeruginosa* isolates from the National Reference Center (NRC) and two external centers (Center 1 and Center 2). In isolates harboring more than one carbapenemase gene, each gene was counted individually. Negative control isolates without detectable carbapenemase genes are reported separately.

### Diagnostic performance

Regarding the detection or exclusion of carbapenemase production, mzCIM and IC-4000 achieved perfect sensitivity (100%) with high specificity (94.2% and 87.0%), resulting in the highest overall performance, closely followed by sCIM. mCIM showed lower sensitivity (93.8%) but second-highest specificity among all tests (96.1%). EDTA-based assays were highly sensitive but less specific, whereas Carba NP showed the opposite pattern (Table 2). Predictive values revealed consistently high NPVs across assays, while PPVs varied substantially between cohorts, particularly in the low-prevalence setting (Table 3).

**TABLE 2:** Test performance of seven phenotypic carbapenemase detection tests in *P. aeruginosa* isolates for 113 CP-PA and 207 nCP-PA^*a*^

Test	TP	FN	TN	FP	Sensitivity % (95% CI)	Specificity % (95% CI)	Indeterminate %	Youden index
mzCIM	113	0	195	12	100 (96.7–100)	94.2 (90.1–96.7)	0	0.94
sCIM	112	1	192	15	99.1 (95.2–99.8)	92.8 (88.4–95.6)	6.2	0.92
mCIM	106	7	199	8	93.8 (87.8–97.0)	96.1 (92.6–98.0)	0.9	0.90
EDTA-750	109	4	180	27	96.5 (91.3–98.6)	87.0 (81.7–90.9)	–	0.83
EDTA-930	111	2	165	42	98.2 (93.8–99.5)	79.7 (73.7–84.6)	–	0.78
IC-4000	113	0	180	27	100 (96.7–100)	87.0 (81.7–90.9)	–	0.87
Carba NP	97	16	205	2	85.8 (78.2–91.1)	99.0 (96.5–99.7)	4.4	0.85

^*a*^ mzCIM, modified zinc-supplemented carbapenem inactivation method; sCIM, simplified carbapenem inactivation method; mCIM, modified carbapenem inactivation method; IC-4000, cloxacillin added test; EDTA-750 and EDTA-930, EDTA added test. TP, true positive; FN, false negative; TN, true negative; FP, false positive. For the calculation of sensitivity and specificity, indeterminate results were counted as negative. Numbers in parentheses indicate the 95% confidence interval.

**TABLE 3:** Positive and negative predictive values of phenotypic carbapenemase detection assays overall and by cohort^*a*^

Test	Overall *n* = 320		NRC *n* = 177		Center 1 *n* = 74		Center 2 *n* = 69	
	PPV % (95% CI)	NPV % (95% CI)	PPV % (95% CI)	NPV (95% CI)	PPV (95% CI)	NPV (95% CI)	PPV (95% CI)	NPV (95% CI)
mzCIM	90.4 (84–94)	**100.0** **(98–100)**	93.8 (87–97**)**	**100.0** **(95–100)**	78.3 (58–90**)**	**100.0** **(93–100)**	80.0 (38–96)	**100.0** **(94–100)**
sCIM	88.2 (81–92)	99.5 (97–100)	91.8 (85–96)	98.7 (93–100)	75.0 (55–88)	**100.0** **(93–100)**	80.0 (38–96)	**100.0** **(94–100)**
mCIM	93.0 (87–96)	96.6 (93–98)	**95.5** **(89–98)**	93.2 (86–97)	81.0 (60–92)	98.1 (90–100)	**100.0** **(51–100)**	100.0 (94–100**)**
EDTA-750	80.1 (73–86)	97.8 (95–99)	90.6 (83–95)	95.1 (88–98)	81.8 (62–93)	**100.0** **(93–100)**	22.2 (9–45)	**100.0** **(93–100)**
EDTA-930	72.5 (65–79)	98.8 (96–100)	85.6 (78–91)	97.3 (91–99)	72.0 (52–86)	**100.0** **(93–100)**	16.7 (67–36)	**100.0** **(92–100)**
IC4000	80.7 (73–86)	**100.0** **(98–100)**	94.8 (88–98)	**100.0** **(95–100)**	75.0 (55–88)	**100.0** **(93–100)**	20.0 (8–42)	**100.0** **(93–100)**
Carba NP	**98.0** **(93–99)**	92.8 (89–96)	97.6 (92–99)	89.4 (82–94)	**100.0** **(76–100)**	90.3 (81–96)	**100.0** **(51–100)**	**100.0** **(94–100)**

^*a*^ Positive predictive value (PPV) and negative predictive value (NPV) with 95% confidence intervals for each assay in the overall dataset and in the three study cohorts. Best result per cohort in bold. As the study cohorts represent enriched, non-representative populations rather than real-world prevalence conditions, PPV and NPV values are cohort-specific and cannot be extrapolated to routine clinical settings.

False-positive results among CIM-based tests were observed predominantly in the same isolates. The only common feature among these isolates was the presence of OXA-like β-lactamases; however, no specific OXA-type was consistently associated with false-positive results. Most indeterminate results were, in descending order, for sCIM (*n* = 20), Carba NP (*n* = 14), and mCIM (*n* = 3). For the CIM-based tests, these were carbapenemase-negative isolates, whereas for Carba NP, indeterminate results were observed in isolates producing various carbapenemases (VIM, NDM, GIM). No pattern regarding the resistome could be identified (Table S1).

### Pairwise comparisons

After FDR adjustment, Carba NP showed significantly lower sensitivity than mzCIM, sCIM, EDTA-930, and IC-4000 (all *P* ~ FDR ~ <0.05); no other sensitivity differences reached significance. Regarding specificity, Carba NP significantly outperformed EDTA-930, EDTA-750, and IC-4000, and mCIM showed significantly higher specificity than both EDTA-based tests (all *P* ~ FDR ~ <0.05).

### ROC analysis and optimal cut-off determination

**Fig 1:**
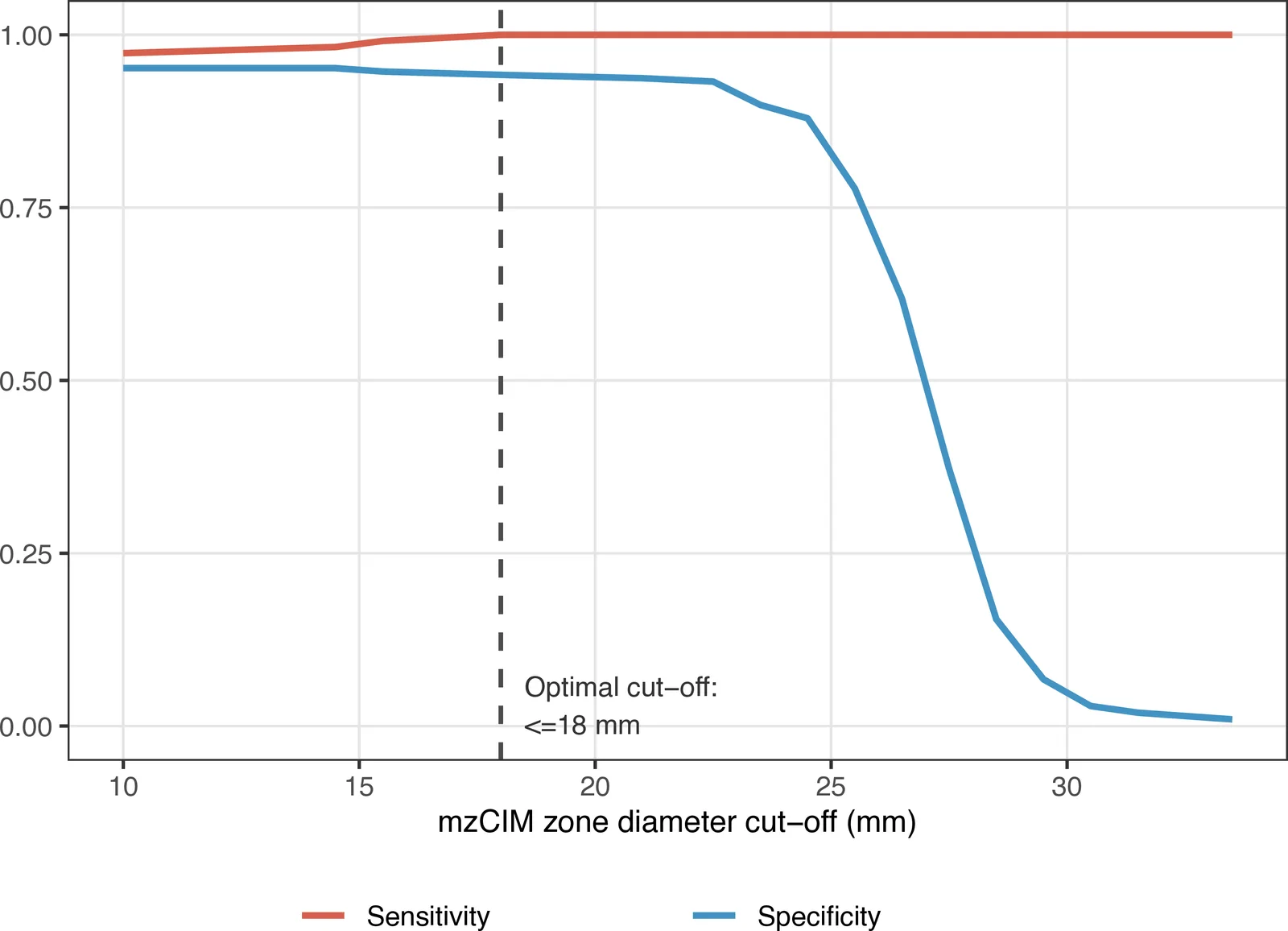
ROC analysis of the mzCIM test: determination of the optimal inhibition zone diameter threshold for carbapenemase detection in *P. aeruginosa*. Diagnostic performance of the mzCIM test across inhibition zone diameter cut-off values. The red line shows sensitivity and the blue line shows specificity at each possible cut-off threshold. Dashed vertical line = optimal cut-off.

**Fig 2:**
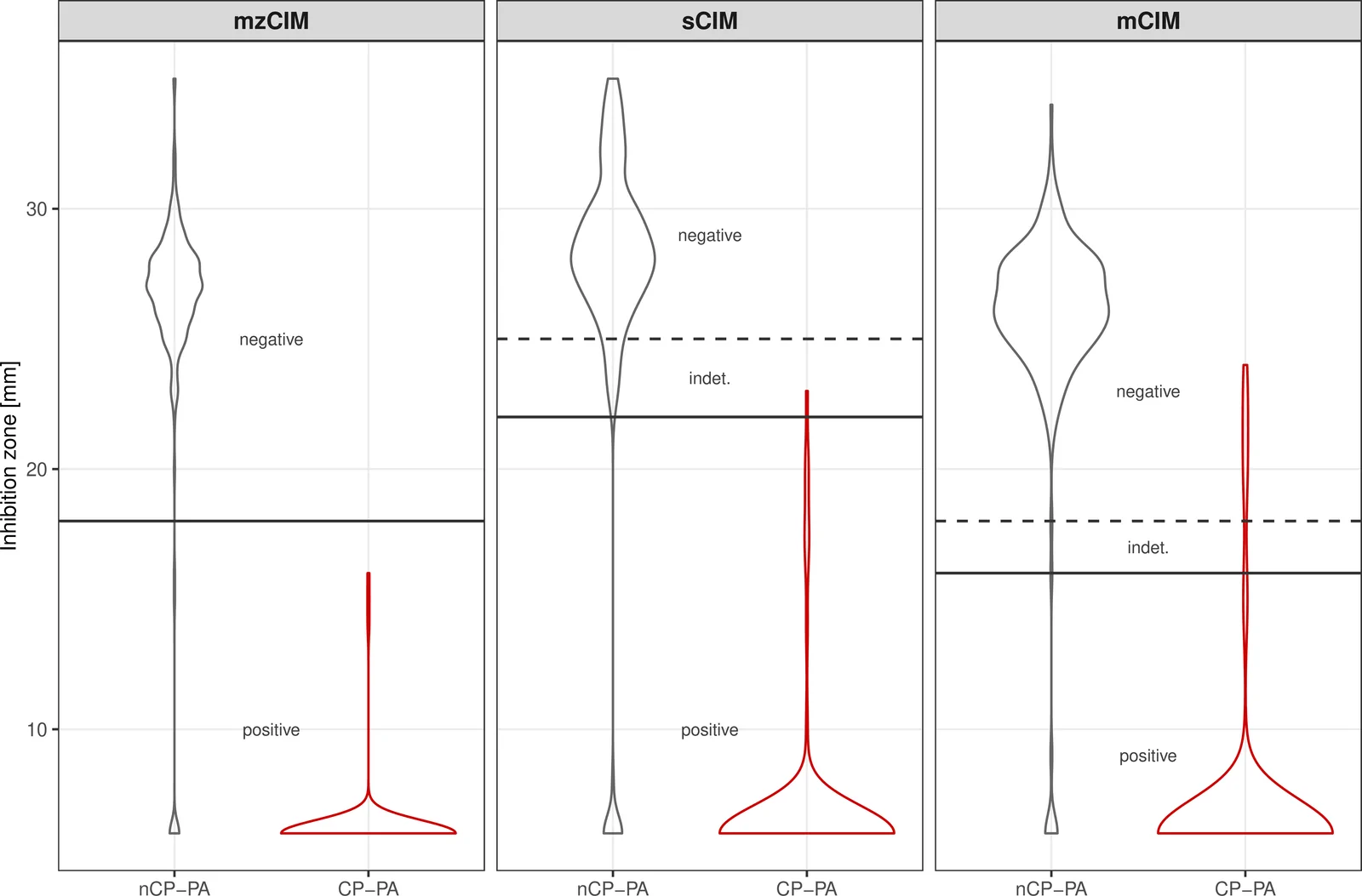
Violin plots of inhibition zones for all CIM-based tests. Distribution of inhibition zone diameters for the mzCIM, sCIM, and mCIM tests in CP-PA and nCP-PA. Horizontal lines indicate established interpretation thresholds: solid lines denote the positive cut-off, dashed lines the upper boundary of the indeterminate zone. *N* = 320 isolates (113 CP-PA, 207 nCP-PA).

ROC analysis of the mzCIM test demonstrated excellent discriminatory performance (AUC 0.975, 95% CI 0.960–0.990) (Fig. 1). The Youden index identified an optimal cut-off of ≤18 mm, at which the test achieved a sensitivity of 100% and a specificity of 94.2% (PPV 90.4%, NPV 100%). No carbapenemase-producing isolate was missed at this threshold (FN = 0). All isolates with inhibition zone diameters >18 mm were carbapenemase-negative, with no overlap between the two groups in this range. A clinically relevant indeterminate zone was not identified (Fig. 2).

ROC analysis of false-positive results in EDTA-based tests and IC-4000: exploratory ROC analyses were performed to assess whether baseline imipenem inhibition zone diameters could help to discriminate false-positive results among carbapenemase-negative isolates.

For the EDTA-based assays, imipenem zone diameter showed no discriminatory ability for predicting false-positive results (AUC 0.193 for EDTA-750 and 0.205 for EDTA-930).

**Fig 3:**
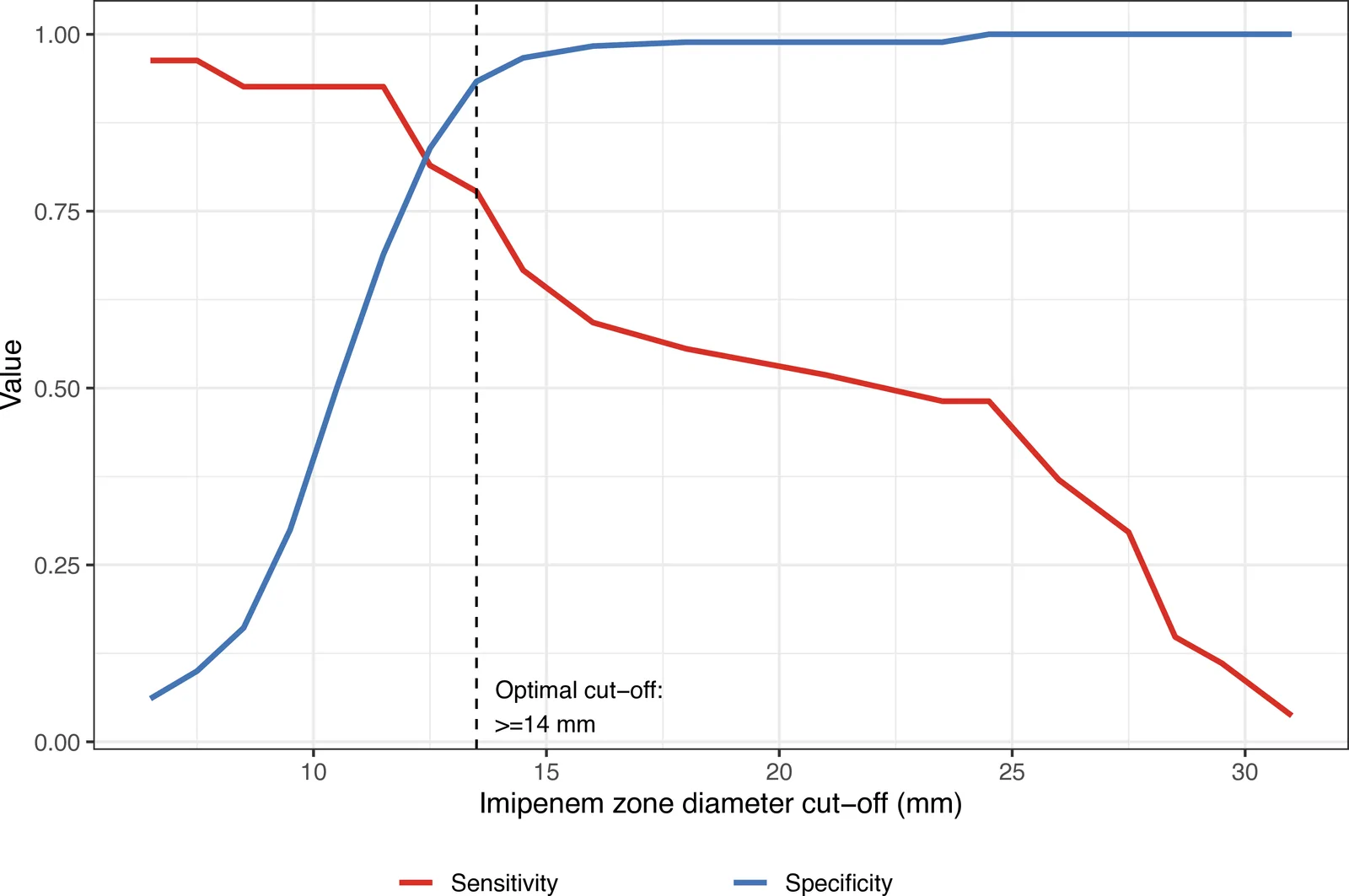
ROC analysis of imipenem inhibition zone diameter as a predictor of false-positive IC-4000 results. ROC curve evaluating the imipenem inhibition zone diameter as a predictor of false-positive IC-4000 results among carbapenemase-negative isolates (*n* = 207). The AUC was 0.893 (95% CI: 0.799–0.986), indicating strong discriminatory ability. The optimal cut-off identified by the Youden criterion was ≥14 mm (sensitivity 77.8%, specificity 93.3%), above which the risk of false-positive IC-4000 results was substantially increased. The diagonal dashed line represents the line of no discrimination.

In contrast, for the IC-4000 assay, the imipenem inhibition zone diameter showed strong discriminatory performance for predicting false-positive results among carbapenemase-negative isolates (AUC 0.893, 95% CI 0.799–0.986). The Youden-optimal cut-off was identified at ≥14 mm, corresponding to a cut-off-sensitivity of 77.8% and a specificity of 93.3% (Fig. 3).

## DISCUSSION

This study systematically evaluated the diagnostic performance of seven phenotypic carbapenemase detection assays for *P. aeruginosa* using isolate collections from three German centers deliberately composed to represent high, intermediate, and low prevalence of carbapenemase-producing isolates.

While CIM-based assays and the Carba NP test directly demonstrate the hydrolytic activity of carbapenemases on carbapenems, the I-C4000 test follows a different principle by differentiating whether carbapenem resistance is attributable to hyperproduction of chromosomal AmpC or to carbapenemase production. Likewise, EDTA-based assays are specific for the detection of MBLs, but do not detect carbapenemases belonging to other Ambler classes.

In the present study, however, this limitation had only a minor impact, as only two isolates produced a non-MBL carbapenemase (Table S1). Nevertheless, the choice and interpretation of these phenotypic assays should always be considered in the context of the local epidemiology and the expected distribution of carbapenem resistance mechanisms.

Among the evaluated assays, CIM-based methods, particularly mzCIM and sCIM, demonstrated the most favorable overall balance between sensitivity and specificity, reflected in the highest Youden indices. mCIM showed a comparable performance profile, albeit with slightly lower sensitivity. These findings support previous observations that CIM-based assays represent robust and broadly applicable phenotypic screening methods for carbapenemase detection in *P. aeruginosa* (31). Pairwise comparisons confirmed that the sensitivity advantage of mzCIM and sCIM over Carba NP was statistically significant, whereas differences among the CIM-based methods themselves were not.

Reduced sensitivity of the Carba NP test for non-fermenting gram-negative bacteria, including *P. aeruginosa*, has been reported previously (32–34). In the cohort-stratified analyses, sensitivity estimates varied considerably. However, the small numbers of carbapenemase-positive isolates in Center 1 and particularly Center 2 limit the interpretability of these cohort-level differences.

The EDTA-based assays showed high sensitivity but reduced specificity in the overall analysis. False-positive results were significantly associated with smaller imipenem inhibition zone diameters among carbapenemase-negative isolates, consistent with the known membrane-destabilizing effects of EDTA on *P. aeruginosa*. In isolates with already reduced carbapenem susceptibility, EDTA-mediated outer membrane disruption may enhance growth inhibition and thereby falsely suggest metallo-β-lactamase activity (35). ROC analysis confirmed that imipenem zone diameter alone does not reliably discriminate false-positive EDTA results, indicating that although the association is statistically significant, its predictive value is limited.

In contrast, false-positive results with IC-4000 were associated with larger imipenem inhibition zones, and zone diameters showed strong discriminatory performance in distinguishing false from true positives (AUC 0.893). ROC analysis identified an optimal cut-off of 14 mm for the baseline imipenem inhibition zone diameter, with zone diameters ≥14 mm being associated with an increased risk of false-positive IC-4000 results. Exclusion of isolates with imipenem inhibition zones ≥14 mm markedly improved specificity from 87.0% to 96.6%, while sensitivity remained unchanged at 100%. This improvement was primarily driven by the removal of a large proportion of false-positive IC-4000 results, as 21 of 27 false positives (77.8%) occurred in isolates with inhibition zones ≥14 mm. However, this approach would also have excluded four true-positive isolates (GIM-1, GES-5, DIM-1, and IMP-1), representing potentially missed carbapenemase producers. Accordingly, when accounting for this preselection step, the effective sensitivity decreases from 100% to 96.5% (109/113). In contrast, 33 true-negative isolates would have been correctly spared from unnecessary testing, highlighting a trade-off between improved specificity and the risk of missing clinically relevant cases.

To our knowledge, mzCIM was assessed for detection of carbapenemases in *P. aeruginosa* for the first time in this study. Cut-off values have previously been established for Enterobacterales, where inhibition zone diameters of ≤18 mm indicate carbapenemase production, 19–20 mm are considered indeterminate, and ≥21 mm are classified as negative (25, 26). Notably, the introduction of an indeterminate zone as defined for Enterobacterales was not necessary for *P. aeruginosa* in our data set. All isolates with inhibition zone diameters >18 mm were carbapenemase-negative, and no carbapenemase-positive isolate exceeded this threshold. It should be noted, however, that thresholds were determined using the present data set and these values should be externally validated in future studies.

The practical implications of sensitivity and specificity trade-offs depend strongly on the epidemiological context. In settings with a high proportion of carbapenemase-producing isolates, false-negative results are of particular concern, as they may lead to missed carbapenemase producers with potential consequences for infection control. Conversely, in settings with lower carbapenemase frequencies, false-positive results become more relevant because they reduce the positive predictive value and may trigger unnecessary confirmatory testing or infection control measures.

This pattern was also reflected in the predictive values shown in Table 3. While NPVs remained consistently high for the most sensitive assays across cohorts, PPVs declined markedly in the low-prevalence cohort, underlining that the practical utility of a positive result is strongly context-dependent.

This study focuses on phenotypic methods for carbapenemase detection. Compared with assays that allow the differentiation of carbapenemases, such as immunochromatographic (ICT) and PCR-based assays, phenotypic tests are more cost-effective and can detect carbapenemases that are not included in most ICTs or PCR panels. They can therefore serve as a valuable complement to molecular diagnostics. However, despite these advantages, their routine implementation may be limited by regulatory requirements, including the European *In Vitro* Diagnostic Regulation. Nevertheless, rare carbapenemases, including those identified in our cohort (e.g., GIM, FIM, and DIM), are not detected by most commercially available methods. Therefore, an initial differentiation test, such as an ICT, may be used for the rapid identification of the most common carbapenemase families. In isolates with negative ICT results but persistent suspicion of carbapenemase production, a phenotypic activity assay should subsequently be performed to detect uncommon or emerging carbapenemases that would otherwise remain undetected.

Several limitations should be considered when interpreting these findings. Isolates were from a single country, and performance might be different in other epidemiological situations. The isolate collections were deliberately assembled to represent differing proportions of carbapenemase-producing isolates rather than reflecting consecutive clinical isolates from defined populations. Consequently, predictive values derived from this data set cannot be directly extrapolated to routine clinical settings. In addition, the limited number of carbapenemase-positive isolates in Center 1 and Center 2 restricts the reliability of cohort-level performance estimates. However, this study comprised a large set of molecularly characterized isolates and represents one of the most comprehensive and systematically conducted comparisons of seven different assays to date.

### Conclusion

CIM-based assays, particularly mzCIM and sCIM, demonstrated the most favorable balance between sensitivity and specificity among the evaluated phenotypic methods, representing the most robust screening approach for carbapenemase detection in *P. aeruginosa*. Their high negative predictive values indicate that these assays are particularly effective for ruling out carbapenemase production. Furthermore, both assays are cost-effective, simple to perform, and can be readily implemented in any routine microbiology laboratory. Widespread adoption of these methods could facilitate a better understanding of the true epidemiology of carbapenemase-producing *P. aeruginosa*.

## Data Availability

The data sets generated during and/or analyzed during the current study are available from the corresponding author on reasonable request. Raw sequencing data are available in the Sequence Read Archive (SRA) of the National Center for Biotechnology Information under BioProject accession number PRJNA1459623.

## SUPPLEMENTAL MATERIAL

Table S1 jcm.00714-26-s0001.docx Characterization of carbapenemase-producing isolates and evaluation of β-lactam susceptibility.

ASM does not own the copyrights to Supplemental Material that may be linked to, or accessed through, an article. The authors have granted ASM a non-exclusive, world-wide license to publish the Supplemental Material files. Please contact the corresponding author directly for reuse.
