# Supplementary material for: Performance of seven carbapenemase detection assays in Pseudomonas aeruginosa across different epidemiological settings: a multicenter cross-sectional study
Source: J Clin Microbiol. 2026 Sep 17;64(10):e00714-26. doi: 10.1128/jcm.00714-26 (PMC13712849; doi:10.1128/jcm.00714-26)
Supplement: Supplementary file 1 [file jcm.00714-26-s0001.docx]

Table S1 Species distribution, carbapenemase detection by phenotypic methods, and antimicrobial susceptibility profiles of Pseudomonas aeruginosa isolates

|  |  |  |  |  |  | **MIC (mg/L)** | | **Phenotypic carbapenemase detection test results** | | | | | | |
| --- | --- | --- | --- | --- | --- | --- | --- | --- | --- | --- | --- | --- | --- | --- |
| **Strain** | **Species** | **Accession-Number** | **Center** | **Carbapenemase(s)** | **Other**  **β-lactamases** | **IMI** | **MER** | **mzCIM result** | **sCIM**  **result** | **mCIM result** | **Δd EDTA 750 µg result** | **Δd EDTA 930 µg result** | **Δd IC-4000 result** | **Carba NP result** |
| PSA-1 | PSA | *SAMN57559930* | NRC | *bla*_VIM-2_ | *bla*_OXA-395_, *bla*_PDC-3_ | ≥ 16 | ≥ 16 | 6 | 6 | 6 | 9 | 10 | 0 | pos |
| PSA-2 | PSA | *SAMN57559931* | NRC | *bla*_VIM-2_ | *bla*_OXA-395_, *bla*_PDC-3_ | ≥ 16 | ≥ 16 | 6 | 6 | 6 | 8 | 9 | 0 | pos |
| PSA-3 | PSA | *SAMN57559932* | NRC | negativ | *bla*_OXA-1245_, *bla*_PDC-24_ | 2 | 1 | 27 | 30 | 25 | 4 | 4 | 4 FP | neg |
| PSA-4 | PSA | *SAMN57559933* | NRC | *bla*_VIM-2_ | *bla*_LCR-1_, *bla*_OXA-395_, *bla*_PDC-3_ | ≥ 16 | ≥ 16 | 6 | 6 | 6 | 12 | 12 | 0 | pos |
| PSA-5 | PSA | *SAMN57559934* | NRC | *bla*_VIM-2_ | *bla*_OXA-395_, *bla*_PDC-3_ | ≥ 16 | ≥ 16 | 6 | 20 | 22 FN | 13 | 14 | 0 | **neg** |
| PSA-6 | PSA | *SAMN57559935* | NRC | *bla*_VIM-2_ | *bla*_OXA-395_, *bla*_PDC-3_ | ≥ 16 | ≥ 16 | 6 | 6 | 6 | 11 | 12 | 0 | pos |
| PSA-7 | PSA | *SAMN57559936* | NRC | *bla*_VIM-2_ | *bla*_OXA-395_, *bla*_PDC-3_ | ≥ 16 | ≥ 16 | 6 | 6 | 6 | 12 | 13 | 0 | pos |
| PSA-8 | PSA | *SAMN57559937* | NRC | *bla*_VIM-2_ | *bla*_OXA-395_, *bla*_PDC-3_ | ≥ 16 | ≥ 16 | 6 | 6 | 6 | 10 | 12 | 0 | pos |
| PSA-9 | PSA | *SAMN57559938* | NRC | *bla*_VIM-2_ | *bla*_OXA-395_, *bla*_PDC-3_ | ≥ 16 | ≥ 16 | 6 | 6 | 6 | 12 | 13 | 0 | pos |
| PSA-10 | PSA | *SAMN57559939* | NRC | *bla*_VIM-2_ | *bla*_OXA-395_, *bla*_PDC-3_ | ≥ 16 | ≥ 16 | 6 | 6 | 6 | 13 | 14 | 0 | pos |
| PSA-11 | PSA | *SAMN57559940* | NRC | *bla*_VIM-2_ | *bla*_OXA-395_, *bla*_PDC-3_ | ≥ 16 | ≥ 16 | 6 | 6 | 6 | 10 | 11 | 0 | pos |
| PSA-12 | PSA | *SAMN57559941* | NRC | *bla*_VIM-2_ | *bla*_OXA-395_, *bla*_PDC-3_ | ≥ 16 | 8 | 6 | 6 | 6 | 15 | 16 | 3 | pos |
| PSA-13 | PSA | *SAMN57559942* | NRC | *bla*_VIM-2_ | *bla*_OXA-395_, *bla*_PDC-3_ | ≥ 16 | ≥ 16 | 6 | 6 | 6 | 11 | 13 | 0 | pos |
| PSA-14 | PSA | *SAMN57559943* | NRC | *bla*_VIM-2_ | *bla*_OXA-395_, *bla*_PDC-3_ | ≥ 16 | ≥ 16 | 6 | 6 | 6 | 10 | 10 | 0 | pos |
| PSA-15 | PSA | *SAMN57559944* | NRC | *bla*_VIM-2_ | *bla*_OXA-395_, *bla*_PDC-3_ | ≥ 16 | ≥ 16 | 6 | 6 | 6 | 10 | 11 | 0 | pos |
| PSA-16 | PSA | *SAMN57559945* | NRC | *bla*_VIM-2_ | *bla*_OXA-395_, *bla*_PDC-3_ | ≥ 16 | ≥ 16 | 14 | 6 | 20 FN | 10 | 11 | 0 | **neg** |
| PSA-17 | PSA | *SAMN57559946* | NRC | *bla*_VIM-2_ | *bla*_OXA-395_, *bla*_PDC-3_ | ≥ 16 | ≥ 16 | 6 | 6 | **16** | 11 | 12 | 0 | pos |
| PSA-18 | PSA | *SAMN57559947* | NRC | *bla*_VIM-2_ | *bla*_OXA-395_, *bla*_PDC-3_ | ≥ 16 | ≥ 16 | 6 | 6 | 6 | 10 | 11 | 0 | pos |
| PSA-19 | PSA | *SAMN57559948* | NRC | *bla*_VIM-2_ | *bla*_OXA-395_, *bla*_PDC-3_ | ≥ 16 | ≥ 16 | 6 | 6 | 6 | 11 | 12 | 0 | pos |
| PSA-20 | PSA | *SAMN57559949* | NRC | *bla*_VIM-2_ | *bla*_OXA-395_, *bla*_PDC-3_ | ≥ 16 | ≥ 16 | 6 | 6 | 6 | 11 | 13 | 0 | pos |
| PSA-21 | PSA | *SAMN57559950* | NRC | *bla*_GIM-1_ | *bla*_OXA-488_, *bla*_PDC-35_ | ≥ 16 | ≥ 16 | 6 | 6 | 6 | 12 | 13 | 0 | pos |
| PSA-22 | PSA | *SAMN57559951* | NRC | *bla*_VIM-2_ | *bla*_OXA-395_, *bla*_PDC-3_ | ≥ 16 | ≥ 16 | 6 | 6 | 6 | 11 | 12 | 0 | pos |
| PSA-23 | PSA | *SAMN57559952* | NRC | *bla*_VIM-2_ | *bla*_OXA-395_, *bla*_PDC-3_ | ≥ 16 | ≥ 16 | 6 | 6 | 6 | 11 | 12 | 0 | pos |
| PSA-24 | PSA | *SAMN57559953* | NRC | *bla*_VIM-2_ | *bla*_OXA-395_, *bla*_PDC-3_ | ≥ 16 | ≥ 16 | 6 | 6 | 6 | 11 | 12 | 0 | pos |
| PSA-25 | PSA | *SAMN57559954* | NRC | *bla*_VIM-2_ | *bla*_OXA-395_, *bla*_PDC-3_ | ≥ 16 | 4 | 6 | 6 | 6 | 16 | 17 | 4 | pos |
| PSA-26 | PSA | *SAMN57559955* | NRC | *bla*_VIM-2_ | *bla*_OXA-395_, *bla*_PDC-3_ | ≥ 16 | 4 | 6 | **23** | 6 | 16 | 17 | 5 | pos |
| PSA-27 | PSA | *SAMN57559956* | NRC | *bla*_VIM-2_ | *bla*_OXA-395_, *bla*_PDC-68_ | ≥ 16 | ≥ 16 | 6 | 6 | 6 | 6 | 7 | 0 | pos |
| PSA-28 | PSA | *SAMN57559957* | NRC | *bla*_VIM-2_ | *bla*_OXA-395_, *bla*_PDC-3_ | ≥ 16 | ≥ 16 | 6 | 6 | 6 | 10 | 11 | 0 | pos |
| PSA-29 | PSA | *SAMN57559958* | NRC | *bla*_VIM-2_ | *bla*_OXA-395_, *bla*_PDC-3_ | ≥ 16 | 8 | 6 | 6 | 6 | 13 | 14 | 3 | pos |
| PSA-30 | PSA | *SAMN57559959* | NRC | *bla*_VIM-2_ | *bla*_OXA-395_, *bla*_PDC-3_ | ≥ 16 | ≥ 16 | 6 | 6 | 6 | 8 | 9 | 0 | pos |
| PSA-31 | PSA | *SAMN57559960* | NRC | *bla*_VIM-2_ | *bla*_OXA-395_, *bla*_PDC-3_ | ≥ 16 | ≥ 16 | 6 | 6 | 6 | 7 | 8 | 0 | pos |
| PSA-32 | PSA | *SAMN57559961* | NRC | *bla*_VIM-2_ | *bla*_OXA-395_, *bla*_PDC-3_ | ≥ 16 | ≥ 16 | 6 | 6 | 15 | 7 | 8 | 0 | pos |
| PSA-33 | PSA | *SAMN57559962* | NRC | *bla*_VIM-2_ | *bla*_OXA-395_, *bla*_PDC-3_ | ≥ 16 | ≥ 16 | 6 | 6 | 6 | 7 | 8 | 0 | pos |
| PSA-34 | PSA | *SAMN57559963* | NRC | *bla*_VIM-2_ | *bla*_OXA-395_, *bla*_PDC-3_ | ≥ 16 | ≥ 16 | 6 | 6 | 6 | 7 | 8 | 0 | pos |
| PSA-35 | PSA | *SAMN57559964* | NRC | *bla*_VIM-2_ | *bla*_OXA-395_, *bla*_PDC-3_ | ≥ 16 | ≥ 16 | 6 | 6 | 6 | 7 | 8 | 0 | pos |
| PSA-36 | PSA | *SAMN57559965* | NRC | *bla*_VIM-2_ | *bla*_OXA-395_, *bla*_PDC-3_ | ≥ 16 | ≥ 16 | 6 | 6 | 6 | 8 | 9 | 0 | pos |
| PSA-37 | PSA | *SAMN57559966* | NRC | *bla*_VIM-2_ | *bla*_OXA-395_, *bla*_PDC-3_ | ≥ 16 | ≥ 16 | 6 | 6 | 23 FN | 7 | 8 | 0 | **neg** |
| PSA-38 | PSA | *SAMN57559967* | NRC | *bla*_VIM-2_ | *bla*_OXA-395_, *bla*_PDC-3_ | ≥ 16 | ≥ 16 | 6 | 6 | 6 | 18 | 19 | 4 | pos |
| PSA-39 | PSA | *SAMN57559968* | NRC | *bla*_IMP-7_ | *bla*_OXA-2_, *bla*_OXA-846_, *bla*_PDC-11_ | ≥ 16 | ≥ 16 | 6 | 6 | 6 | 8 | 11 | 0 | pos |
| PSA-40 | PSA | *SAMN57559969* | NRC | *bla*_IMP-22_ | *bla*_OXA_, *bla*_OXA-905_, *bla*_PDC-8_ | ≥ 16 | ≥ 16 | 6 | 6 | 6 | 8 | 10 | 5 | pos |
| PSA-41 | PSA | *SAMN57559970* | NRC | *bla*_GIM-1_ | *bla*_OXA-905_, *bla*_PDC-8_ | ≥ 16 | ≥ 16 | 6 | 6 | 6 | 8 | 11 | 0 | pos |
| PSA-42 | PSA | *SAMN57559971* | NRC | negativ | *bla*_OXA-2_, *bla*_OXA-488_, *bla*_PDC-35_ | ≥ 16 | ≥ 16 | 6 FP | 28 | **16** | 4 | 4 | 10 | *neg* |
| PSA-43 | PSA | *SAMN57559972* | NRC | *bla*_VIIM-1_ | *bla*_OXA-488_, *bla*_PDC-35_ | ≥ 16 | ≥ 16 | 6 | 6 | 6 | 10 | 11 | 0 | **neg** |
| PSA-44 | PSA | *SAMN57559973* | NRC | *bla*_GIM-1_ | *bla*_OXA-905_, *bla*_PDC-8_ | ≥ 16 | ≥ 16 | 6 | 6 | 6 | 10 | 11 | 0 | pos |
| PSA-45 | PSA | *SAMN57559974* | NRC | *bla*_VIM-4_ | *bla*_OXA-1127_, *bla*_PDC-5_ | ≥ 16 | ≥ 16 | 6 | 6 | 6 | 9 | 10 | 0 | pos |
| PSA-46 | PSA | *SAMN57559975* | NRC | *bla*_GIM-1_ | *bla*_OXA-1035_, *bla*_PDC-19a_ | ≥ 16 | ≥ 16 | 6 | 6 | 6 | 12 | 12 | 4 | pos |
| PSA-47 | PSA | *SAMN57559976* | NRC | *bla*_GIM-1_ | *bla*_OXA-905_, *bla*_PDC-8_ | ≥ 16 | ≥ 16 | 6 | 6 | 6 | 10 | 10 | 0 | pos |
| PSA-48 | PSA | *SAMN57559977* | NRC | *bla*_VIM-2_ | *bla*_OXA-395_, *bla*_PDC-3_ | ≥ 16 | ≥ 16 | 6 | 6 | 6 | 10 | 11 | 0 | pos |
| PSA-49 | PSA | *SAMN57559978* | NRC | *bla*_NDM-1_ | *bla*_OXA-395_, *bla*_PDC-16_ | ≥ 16 | ≥ 16 | 6 | 6 | 6 | 9 | 9 | 0 | pos |
| PSA-50 | PSA | *SAMN57559979* | NRC | *bla*_VIM-2_ | *bla*_OXA-1127_, *bla*_PDC-5_ | ≥ 16 | ≥ 16 | 6 | 6 | 6 | 8 | 10 | 0 | pos |
| PSA-51 | PSA | *SAMN57559980* | NRC | *bla*_VIM-2_ | *bla*_OXA-488_, *bla*_PDC-35_ | ≥ 16 | ≥ 16 | 6 | 6 | 6 | 12 | 13 | 0 | pos |
| PSA-52 | PSA | *SAMN57559981* | NRC | *bla*_VIM-2_ | *bla*_OXA-486_, *bla*_PDC-3_ | ≥ 16 | ≥ 16 | 6 | 6 | 6 | 8 | 10 | 0 | pos |
| PSA-53 | PSA | *SAMN57559982* | NRC | *bla*_IMP-1_ | *bla*_OXA-488_, *bla*_PDC-35_ | ≥ 16 | ≥ 16 | 6 | 6 | 6 | 10 | 13 | 0 | pos |
| PSA-54 | PSA | *SAMN57559983* | NRC | *bla*_GIM-1_ | *bla*_OXA-2_, *bla*_OXA-1127_, *bla*_PDC-5_ | ≥ 16 | ≥ 16 | 6 | 6 | 6 | 9 | 9 | 0 | pos |
| PSA-55 | PSA | *SAMN57559984* | NRC | *bla*_VIM-1_ | *bla*_OXA-494_, *bla*_PDC-5_ | ≥ 16 | ≥ 16 | 6 | 6 | 24 FN | 8 | 9 | 0 | pos |
| PSA-56 | PSA | *SAMN57559985* | NRC | *bla*_VIM-2_ | *bla*_OXA-395_, *bla*_PDC-3_ | ≥ 16 | ≥ 16 | 6 | 6 | 6 | 10 | 10 | 0 | pos |
| PSA-57 | PSA | *SAMN57559986* | NRC | *bla*_VIM-2_ | *bla*_OXA-395_, *bla*_PDC-3_ | ≥ 16 | ≥ 16 | 6 | 6 | 6 | 10 | 10 | 0 | pos |
| PSA-58 | PSA | *SAMN57559987* | NRC | *bla*_VIM-2_ | *bla*_OXA-395_, *bla*_PDC-3_ | ≥ 16 | ≥ 16 | 6 | 6 | 6 | 11 | 12 | 0 | pos |
| PSA-59 | PSA | *SAMN57559988* | NRC | *bla*_GIM-1_ | *bla*_OXA-905_, *bla*_PDC-8_ | ≥ 16 | ≥ 16 | 6 | 6 | 6 | 9 | 10 | 0 | pos |
| PSA-60 | PSA | *SAMN57559989* | NRC | *bla*_NDM-1_ | *bla*_PAO_, *bla*_OXA-395_ | ≥ 16 | ≥ 16 | 6 | 6 | 6 | 10 | 11 | 0 | pos |
| PSA-61 | PSA | *SAMN57559990* | NRC | negativ | *bla*_OXA-10_, *bla*_OXA-488_, *bla*_PDC-35_ | ≥ 16 | ≥ 16 | 6 FP | 6 FP | 6 FP | 2 | 3 | 5 FP | pos FP |
| PSA-62 | PSA | *SAMN57559991* | NRC | negativ | *bla*_OXA-488_, *bla*_PAO_, *bla*_OXA-10_ | ≥ 16 | ≥ 16 | 6 FP | 6 FP | 6 FP | 1 | 2 | 6 | pos FP |
| PSA-63 | PSA | *SAMN57559992* | NRC | *bla*_IMP-13_ |  | ≥ 16 | ≥ 16 | 6 | 6 | 6 | 12 | 14 | 2 | pos |
| PSA-64 | PSA | *SAMN57559993* | NRC | *bla*_NDM-1_ | *bla*_PAO_, *bla*_OXA-395_, | ≥ 16 | ≥ 16 | 6 | 6 | 6 | 9 | 11 | 0 | neg |
| PSA-65 | PSA | *SAMN57559994* | NRC | *bla*_NDM-1_ | *bla*_OXA-395_, *bla*_PDC-16_ | ≥ 16 | ≥ 16 | 6 | 6 | 6 | 9 | 11 | 0 | neg |
| PSA-66 | PSA | *SAMN57559995* | NRC | *bla*_GIM-1_ | *bla*_PAO_, *bla*_OXA-488_, *bla*_OXA-485_ | ≥ 16 | ≥ 16 | 6 | 10 | 6 | 9 | 10 | 0 | pos |
| PSA-67 | PSA | *SAMN57559996* | NRC | *bla*_IMP-1_ | *bla*_OXA-10_, *bla*_OXA-488_, *bla*_PDC-12_ | ≥ 16 | ≥ 16 | 6 | 6 | 6 | 12 | 14 | 0 | pos |
| PSA-68 | PSA | *SAMN57559997* | NRC | *bla*_GIM-1_ | *bla*_OXA-905_, *bla*_PDC-8_ | ≥ 16 | ≥ 16 | 6 | 6 | 6 | 12 | 13 | 0 | pos |
| PSA-69 | PSA | *SAMN57559998* | NRC | *bla*_VIM-2_ | *bla*_OXA-395_, *bla*_PDC-3_ | ≥ 16 | ≥ 16 | 6 | 6 | 6 | 9 | 11 | 0 | pos |
| PSA-70 | PSA | *SAMN57559999* | NRC | *bla*_NDM-1_ | *bla*_OXA-395_, *bla*_PDC-16_ | ≥ 16 | ≥ 16 | 16 | 6 | 14 | 8 | 8 | 0 | pos |
| PSA-71 | PSA | *SAMN57560000* | NRC | *bla*_KPC-2_ | *bla*_PAO_ | ≥ 16 | ≥ 16 | 6 | 6 | 6 | 3 | 3 | 0 | pos |
| PSA-72 | PSA | *SAMN57560001* | NRC | *bla*_DIM-1_, *bla*_IMP-1b_ | *bla*_PAO_, *bla*_OXA-10_, *bla*_OXA-488_ | ≥ 16 | ≥ 16 | 6 | 6 | 6 | 6 FN | 7 | 0 | pos |
| PSA-73 | PSA | *SAMN57560002* | NRC | *bla*_GIM-1_ | *bla*_OXA-488_, *bla*_PDC-35_ | ≥ 16 | ≥ 16 | 6 | 6 | 6 | 16 | 18 | 4 | pos |
| PSA-74 | PSA | *SAMN57560003* | NRC | *bla*_GIM-1_ | *bla*_PAO_, *bla*_OXA-488_ | ≥ 16 | ≥ 16 | 6 | 6 | 6 | 10 | 11 | 0 | pos |
| PSA-75 | PSA | *SAMN57560004* | NRC | *bla*_NDM-1_ | *bla*_OXA-395_, *bla*_PDC-16_ | ≥ 16 | ≥ 16 | 6 | 17 | 21 FN | 7 | 8 | 0 | pos |
| PSA-76 | PSA | *SAMN57560005* | NRC | *bla*_IMP-1_ | *bla*_OXA-10_. *bla*_OXA-488_, *bla*_PDC12_ | ≥ 16 | ≥ 16 | 6 | 6 | 6 | 8 | 9 | 0 | pos |
| PSA-77 | PSA | *SAMN57560006* | NRC | *bla*_NDM-1_ | *bla*_OXA-395_, *bla*_PDC-16_ | ≥ 16 | ≥ 16 | 6 | 6 | 6 | 8 | 9 | 0 | pos |
| PSA-78 | PSA | *SAMN57560007* | NRC | *bla*_GIM-1_ | *bla*_OXA-847_, *bla*_PDC-1_ | ≥ 16 | ≥ 16 | 6 | 6 | 6 | 18 | 19 | 0 | pos |
| PSA-79 | PSA | *SAMN57560008* | NRC | *bla*_NDM-1_ | *bla*_OXA-395_, *bla*_PDC-16_ | ≥ 16 | ≥ 16 | 6 | 18 | 6 | 7 | 8 | 0 | pos |
| PSA-80 | PSA | *SAMN57560009* | NRC | *bla*_GES-5_, *bla*_NDM-1_ | *bla*_CTX-M-15_, *bla*_OXA-1_, *bla*_OXA-488_, *bla*_PDC_, *bla*_SHV-11_ | ≥ 16 | ≥ 16 | 6 | 6 | 6 | 0 | -7 | 0 | *neg* |
| PSA-81 | PSA | *SAMN57560010* | NRC | *bla*_FIM-1_ | *bla*_CARB-2_, *bla*_OXA-205_, *bla*_OXA-488_, *bla*_PDC-35_, *bla*_SHV-2A_ | ≥ 16 | ≥ 16 | 6 | 6 | 6 | 10 | 12 | 0 | pos |
| PSA-82 | PSA | *SAMN57560011* | NRC | *bla*_NDM-1_ | *bla*_OXA-395_, *bla*_PDC-16_ | ≥ 16 | ≥ 16 | 6 | 6 | 6 | 10 | 11 | 0 | pos |
| PSA-83 | PSA | *SAMN57560012* | NRC | *bla*_NDM-1_ | *bla*_OXA-10_, *bla*_OXA-846_, *bla*_PDC-11_, *bla*_VEB-9_ | ≥ 16 | ≥ 16 | 6 | 6 | 6 | 10 | 10 | 0 | *neg* |
| PSA-84 | PSA | *SAMN57560013* | NRC | negativ | OXA-10, *bla*_OXA-488_, *bla*_PDC-35_, *bla*_SHV-12_ | ≥ 16 | ≥ 16 | 6 FP | 6 FP | 6 FP | 4 | 5 | 7 | neg |
| PSA-85 | PSA | *SAMN57560014* | NRC | *bla*_NDM-1_ | *bla*_OXA-395_, *bla*_PDC-16_ | ≥ 16 | ≥ 16 | 6 | 6 | 6 | 23 | 24 | 1 | pos |
| PSA-86 | PSA | *SAMN57560015* | NRC | *bla*_IMP-1_ | *bla*_OXA-10_, *bla*_OXA-488_, *bla*_PDC-12_ | ≥ 16 | ≥ 16 | 6 | 6 | 6 | 10 | 10 | 0 | pos |
| PSA-87 | PSA | *SAMN57560016* | NRC | *bla*_GIM-1_ | *bla*_OXA-1127_, *bla*_PDC-5_ | ≥ 16 | ≥ 16 | 6 | 6 | 6 | 8 | 8 | 0 | pos |
| PSA-88 | PSA | *SAMN57560017* | NRC | *bla*_VIM-2_ | *bla*_OXA-395_, *bla*_PDC-3_ | ≥ 16 | ≥ 16 | 6 | 6 | 6 | 9 | 10 | 0 | pos |
| PSA-89 | PSA | *SAMN57560018* | NRC | *bla*_IMP-7_ | *bla*_OXA-2_, *bla*_OXA-846_, *bla*_PDC-11_ | ≥ 16 | ≥ 16 | 6 | 6 | 6 | 8 | 9 | 0 | pos |
| PSA-90 | PSA | *SAMN57560019* | NRC | *bla*_NDM-1_ | *bla*_OXA-395_, *bla*_PDC-16_ | ≥ 16 | ≥ 16 | 6 | 6 | 6 | 11 | 14 | 0 | **neg** |
| PSA-91 | PSA | *SAMN57560020* | NRC | *bla*_IMP-94_ | *bla*_OXA-10_, *bla*_OXA-396_, *bla*_PDC-3_ | ≥ 16 | ≥ 16 | 6 | 6 | 6 | 10 | 12 | 1 | pos |
| PSA-92 | PSA | *SAMN57560021* | NRC | negativ | *bla*_GES-59_, *bla*_OXA-1028_, *bla*_PDC-5_ | ≥ 16 | ≥ 16 | 25 | 28 | 26 | 5 | 9 FP | 3 FP | neg |
| PSA-93 | PSA | *SAMN57560022* | NRC | *bla*_VIM-2_ | *bla*_OXA-396_, *bla*_OXA-520_, *bla*_PDC_ | NA | NA | 6 | 6 | 6 | 23 | 24 | 0 | pos |
| PSA-94 | PSA | *SAMN57560023* | NRC | *bla*_DIM-1_ | *bla*_OXA-851_, *bla*_PDC-120_ | ≥ 16 | ≥ 16 | 6 | 6 | 6 | 9 | 9 | 4 | pos |
| PSA-95 | PSA | *SAMN57560024* | NRC | *bla*_IMP-1_ | *bla*_OXA-10_. *bla*_OXA-488_, *bla*_PDC-12_ | ≥ 16 | ≥ 16 | 6 | 6 | 6 | 13 | 13 | 2 | pos |
| PSA-96 | PSA | *SAMN57560025* | NRC | *bla*_NDM-1_ | *bla*_OXA-395_, *bla*_PDC-16_ | ≥ 16 | ≥ 16 | 6 | 6 | 6 | 10 | 11 | 0 | pos |
| PSA-97 | PSA | *SAMN57560026* | NRC | negativ | *bla*_OXA-677_, *bla*_OXA-846_, *bla*_PDC-11_, *bla*_VEB-9_ | ≥ 16 | ≥ 16 | 23 | 28 | 23 | 4 | 5 | 9 | neg |
| PSA-98 | PSA | *SAMN57560027* | NRC | negativ | *bla*_OXA-395_, *bla*_PDC-30_ | ≥ 16 | ≥ 16 | 24 | 27 | 22 | 1 | 1 | 9 | neg |
| PSA-99 | PSA | *SAMN57560028* | NRC | negativ | *bla*_OXA-904_, *bla*_PDC-60_ | ≥ 16 | ≥ 16 | 24 | 32 | 23 | 4 | 5 | 11 | neg |
| PSA-100 | PSA | *SAMN57560029* | NRC | negativ | *bla*_OXA-847_, *bla*_PDC-24_ | ≥ 16 | ≥ 16 | 24 | 33 | 24 | 2 | 3 | 10 | neg |
| PSA-101 | PSA | *SAMN57560030* | NRC | negativ | *bla*_OXA-1033_, *bla*_PDC-3_ | ≥ 16 | ≥ 16 | 25 | 34 | 24 | 3 | 5 | 10 | neg |
| PSA-102 | PSA | *SAMN57560031* | NRC | negativ | *bla*_OXA-494_, *bla*_PDC-3_ | ≥ 16 | ≥ 16 | 23 | 8 FP | 25 | 3 | 5 | 8 | neg |
| PSA-103 | PSA | *SAMN57560032* | NRC | negativ | *bla*_OXA-396_, *bla*_PDC-5_ | ≥ 16 | ≥ 16 | 20 | 30 | 26 | 8 FP | 9 FP | 9 | neg |
| PSA-104 | PSA | *SAMN57560033* | NRC | negativ | *bla*_OXA-847_, *bla*_PDC-3_ | ≥ 16 | ≥ 16 | 28 | 6 FP | 24 | 5 | 6 | 6 | neg |
| PSA-105 | PSA | *SAMN57560034* | NRC | negativ | *bla*_OXA-486_, *bla*_PDC-3_ | ≥ 16 | ≥ 16 | 28 | 32 | 25 | 7 FP | 8 FP | 7 | neg |
| PSA-106 | PSA | *SAMN57560035* | NRC | negativ | *bla*_PAO_, *bla*_OXA-494_, *bla*_OXA-396_ | ≥ 16 | ≥ 16 | 27 | 34 | 26 | 1 | 3 | 13 | neg |
| PSA-107 | PSA | *SAMN57560036* | NRC | negativ | *bla*_PAO_, *bla*_OXA-494_, *bla*_OXA-396_ | ≥ 16 | ≥ 16 | 26 | 29 | 26 | 7 FP | 7 FP | 13 | neg |
| PSA-108 | PSA | *SAMN57560037* | NRC | negativ | *bla*_PAO_, *bla*_OXA-486_ | ≥ 16 | ≥ 16 | 28 | 33 | 25 | 7 FP | 8 FP | 6 | neg |
| PSA-109 | PSA | *SAMN57560038* | NRC | negativ | *bla*_PAO_ | ≥ 16 | ≥ 16 | 27 | 34 | 25 | 5 | 6 | 13 | neg |
| PSA-110 | PSA | *SAMN57560039* | NRC | negativ | *bla*_PAO_, *bla*_OXA-486_ | ≥ 16 | ≥ 16 | 27 | 35 | 25 | 4 | 4 | 9 | neg |
| PSA-111 | PSA | *SAMN57560040* | NRC | negativ | *bla*_OXA-494_, *bla*_OXA-396_, *bla*_PAO_ | ≥ 16 | ≥ 16 | 27 | 34 | 27 | 2 | 3 | 10 | neg |
| PSA-112 | PSA | *SAMN57560041* | NRC | negativ | *bla*_PAO_, *bla*_OXA-396_, *bla*_OXA-494_ | ≥ 16 | ≥ 16 | 27 | 32 | 26 | 1 | 3 | 13 | neg |
| PSA-113 | PSA | *SAMN57560042* | NRC | negativ | *bla*_OXA-486_, *bla*_PDC-24_ | ≥ 16 | ≥ 16 | 27 | 33 | 26 | 3 | 4 | 9 | neg |
| PSA-114 | PSA | *SAMN57560043* | NRC | negativ | *bla*_PAO_, *bla*_OXA-396_, *bla*_OXA-494_ | ≥ 16 | ≥ 16 | 30 | 32 | 25 | 6 | 8 FP | 14 | neg |
| PSA-115 | PSA | *SAMN57560044* | NRC | negativ | *bla*_PAO_, *bla*_OXA-50_ | ≥ 16 | ≥ 16 | 29 | 32 | 28 | 3 | 3 | 14 | neg |
| PSA-116 | PSA | *SAMN57560045* | NRC | negativ | *bla*_OXA-494_, *bla*_OXA-396_, *bla*_PAO_ | ≥ 16 | ≥ 16 | 27 | 34 | 27 | 9 FP | 9 FP | 16 | neg |
| PSA-117 | PSA | *SAMN57560046* | NRC | negativ | *bla*_PAO_, *bla*_OXA-396_ | ≥ 16 | ≥ 16 | 28 | 34 | 26 | 3 | 4 | 8 | neg |
| PSA-118 | PSA | *SAMN57560047* | NRC | negativ | *bla*_OXA-50_, *bla*_PAO_ | NA | NA | 27 | 35 | 28 | 3 | 4 | 11 | neg |
| PSA-119 | PSA | *SAMN57560048* | NRC | negativ | *bla*_PAO_, *bla*_OXA-486_ | ≥ 16 | ≥ 16 | 28 | 30 | 26 | -4 | -4 | 2 FP | neg |
| PSA-120 | PSA | *SAMN57560049* | NRC | negativ | *bla*_OXA-1026_, *bla*_PDC-39_ | ≥ 16 | ≥ 16 | 26 | 35 | 26 | 1 | 2 | 10 | neg |
| PSA-121 | PSA | *SAMN57560050* | NRC | negativ | *bla*_OXA-395_, *bla*_PDC-30_ | ≥ 16 | ≥ 16 | 26 | 34 | 27 | 2 | 3 | 11 | neg |
| PSA-122 | PSA | *SAMN57560051* | NRC | negativ | *bla*_OXA-10_, *bla*_OXA-846_, *bla*_PDC-11_, *bla*_VEB-9_ | ≥ 16 | ≥ 16 | 28 | 33 | 26 | 6 | 7 FP | 10 | neg |
| PSA-123 | PSA | *SAMN57560052* | NRC | negativ | *bla*_OXA-50_, *bla*_PDC-31_ | 4 | 4 | 27 | 33 | 25 | 4 | 5 | 13 | neg |
| PSA-124 | PSA | *SAMN57560053* | NRC | negativ | *bla*_OXA-905_, *bla*_PDC-8_ | ≥ 16 | ≥ 16 | 30 | 33 | 26 | 1 | 2 | 6 | neg |
| PSA-125 | PSA | *SAMN57560054* | NRC | negativ | *bla*_OXA-488_, *bla*_PDC-34_ | ≥ 16 | ≥ 16 | 28 | 32 | 28 | 7 FP | 8 FP | 10 | neg |
| PSA-126 | PSA | *SAMN57560055* | NRC | negativ | *bla*_OXA-486_, *bla*_PDC-3_ | ≥ 16 | ≥ 16 | 27 | 32 | 27 | 4 | 5 | 10 | neg |
| PSA-127 | PSA | *SAMN57560056* | NRC | negativ | *bla*_OXA-50_, *bla*_PDC-31_ | ≥ 16 | ≥ 16 | 28 | 33 | 27 | 1 | 2 | 9 | neg |
| PSA-128 | PSA | *SAMN57560057* | NRC | negativ | *bla*_OXA-488_, *bla*_PDC-35_ | ≥ 16 | ≥ 16 | 28 | 34 | 28 | 2 | 3 | 13 | neg |
| PSA-129 | PSA | *SAMN57560058* | NRC | negativ | *bla*_OXA-851_, *bla*_PDC-46_ | ≥ 16 | NA | 27 | 33 | 28 | 3 | 4 | 7 | neg |
| PSA-130 | PSA | *SAMN57560059* | NRC | negativ | *bla*_OXA-486_, *bla*_PDC-8_ | ≥ 16 | 8 | 28 | 32 | 28 | 4 | 5 | 11 | neg |
| PSA-131 | PSA | *SAMN57560060* | NRC | negativ | *bla*_OXA-848_, *bla*_PDC-16_ | ≥ 16 | ≥ 16 | 26 | 28 | 28 | 3 | 5 | 8 | neg |
| PSA-132 | PSA | *SAMN57560061* | NRC | negativ | *bla*_OXA-905_, *bla*_PDC-8_ | ≥ 16 | ≥ 16 | 27 | 28 | 28 | 5 | 5 | 10 | neg |
| PSA-133 | PSA | *SAMN57560062* | NRC | negativ | *bla*_OXA-486_, *bla*_PDC-8_ | 4 | 4 | 28 | 30 | 27 | 4 | 5 | 12 | neg |
| PSA-134 | PSA | *SAMN57560063* | NRC | negativ | *bla*_OXA-392_, *bla*_OXA-904_, *bla*_PDC-117_ | ≥ 16 | ≥ 16 | 25 | 29 | 26 | 3 | 4 | 7 | neg |
| PSA-135 | PSA | *SAMN57560064* | NRC | negativ | *bla*_OXA-494_, *bla*_PDC-60_ | ≥ 16 | ≥ 16 | 25 | 29 | 27 | 3 | 3 | 12 | neg |
| PSA-136 | PSA | *SAMN57560065* | NRC | negativ | *bla*_OXA-50_, *bla*_PDC-1_ | 8 | 8 | 28 | 28 | 25 | 4 | 4 | 12 | neg |
| PSA-137 | PSA | *SAMN57560066* | NRC | negativ | *bla*_OXA-1014_, *bla*_PDC-5_ | 8 | ≥ 16 | 28 | 29 | 26 | 3 | 4 | 8 | neg |
| PSA-138 | PSA | *SAMN57560067* | NRC | negativ | *bla*_OXA-847_, *bla*_PDC-24_ | ≥ 16 | ≥ 16 | 26 | 28 | 25 | 3 | 4 | 11 | neg |
| PSA-139 | PSA | *SAMN57560068* | NRC | negativ | *bla*_OXA-396_, *bla*_PDC-3_ | ≥ 16 | 8 | 27 | 32 | 26 | 6 | 6 | 14 | neg |
| PSA-140 | PSA | *SAMN57560069* | NRC | negativ | *bla*_OXA-486_, *bla*_PDC-24_ | ≥ 16 | ≥ 16 | 6 FP | 29 | **18** | 4 | 5 | 13 | neg |
| PSA-141 | PSA | *SAMN57560070* | NRC | negativ | *bla*_OXA-851_, *bla*_PDC-5_ | ≥ 16 | ≥ 16 | 26 | 22 | 23 | 4 | 4 | 12 | neg |
| PSA-142 | PSA | *SAMN57560071* | NRC | negativ | *bla*_OXA-1245_, *bla*_PDC-1_ | ≥ 16 | ≥ 16 | 28 | 28 | 25 | 5 | 6 | 10 | neg |
| PSA-143 | PSA | *SAMN57560072* | NRC | negativ | *bla*_OXA-486_, *bla*_PDC-3_ | ≥ 16 | ≥ 16 | 30 | **24** | 28 | 2 | 3 | 10 | neg |
| PSA-144 | PSA | *SAMN57560073* | NRC | negativ | *bla*_OXA-50_, *bla*_PDC-8_ | 8 | ≥ 16 | 29 | 28 | 25 | 5 | 8 FP | 7 | neg |
| PSA-145 | PSA | *SAMN57560074* | NRC | negativ | *bla*_OXA-904_, *bla*_PDC-60_ | ≥ 16 | ≥ 16 | 29 | **25** | 24 | 2 | 3 | 11 | neg |
| PSA-146 | PSA | *SAMN57560075* | NRC | negativ | *bla*_OXA-1034_, *bla*_PDC-3_ | ≥ 16 | ≥ 16 | 27 | 26 | 27 | 4 | 5 | 14 | neg |
| PSA-147 | PSA | *SAMN57560076* | NRC | negativ | *bla*_OXA-851_, *bla*_PDC-3_ | ≥ 16 | ≥ 16 | 27 | 29 | 27 | 4 | 5 | 12 | neg |
| PSA-148 | PSA | *SAMN57560077* | NRC | negativ | *bla*_OXA-10_, *bla*_OXA-488_, *bla*_PDC-12_ | ≥ 16 | 8 | 6 FP | 29 | 6 FP | 4 | 5 | 11 | neg |
| PSA-149 | PSA | *SAMN57560078* | NRC | negativ | *bla*_OXA_, *bla*_PDC-124_ | ≥ 16 | ≥ 16 | 29 | 28 | 25 | 3 | 4 | 12 | neg |
| PSA-150 | PSA | *SAMN57560079* | NRC | negativ | *bla*_OXA-905_, *bla*_PDC-8_ | ≥ 16 | ≥ 16 | 27 | 27 | 26 | 2 | 5 | 8 | neg |
| PSA-151 | PSA | *SAMN57560080* | NRC | negativ | *bla*_OXA-851_, *bla*_PDC-3_ | ≥ 16 | ≥ 16 | 25 | 27 | 26 | 4 | 4 | 11 | neg |
| PSA-152 | PSA | *SAMN57560081* | NRC | negativ | *bla*_OXA-904_, *bla*_PDC-23_ | ≥ 16 | ≥ 16 | 25 | **23** | 23 | 2 | 3 | 9 | neg |
| PSA-153 | PSA | *SAMN57560082* | NRC | negativ | *bla*_OXA-904_, *bla*_PDC-3_ | ≥ 16 | ≥ 16 | 28 | 27 | 24 | 5 | 6 | 9 | neg |
| PSA-154 | PSA | *SAMN57560083* | NRC | negativ | *bla*_OXA-494_, *bla*_PDC-5_ | ≥ 16 | ≥ 16 | 29 | 6 | 23 | 5 | 6 | 11 | neg |
| PSA-155 | PSA | *SAMN57560084* | NRC | negativ | *bla*_OXA-50_, *bla*_PDC-3_ | ≥ 16 | ≥ 16 | 27 | 28 | 26 | 3 | 3 | 9 | neg |
| PSA-156 | PSA | *SAMN57560085* | NRC | negativ | *bla*_OXA-486_, *bla*_PDC-1_ | ≥ 16 | ≥ 16 | 27 | **24** | 26 | 3 | 4 | 10 | neg |
| PSA-157 | PSA | *SAMN57560086* | NRC | negativ | *bla*_OXA-851_, *bla*_PDC-3_ | ≥ 16 | ≥ 16 | 30 | 26 | 27 | 1 | 2 | 10 | neg |
| PSA-158 | PSA | *SAMN57560087* | NRC | negativ | *bla*_OXA-1_, *bla*_OXA-486_, *bla*_PDC-10_ | ≥ 16 | ≥ 16 | 26 | **25** | 27 | 8 FP | 9 FP | 10 | neg |
| PSA-159 | PSA | *SAMN57560088* | NRC | negativ | *bla*_OXA-904_, *bla*_PDC-3_ | ≥ 16 | ≥ 16 | 25 | 26 | 24 | 6 | 7 FP | 12 | neg |
| PSA-160 | PSA | *SAMN57560089* | NRC | negativ | *bla*_OXA_, *bla*_PDC-5_ | ≥ 16 | ≥ 16 | 25 | 27 | 25 | 3 | 4 | 12 | neg |
| PSA-161 | PSA | *SAMN57560090* | NRC | negativ | *bla*_OXA-905_, *bla*_PDC-8_ | ≥ 16 | ≥ 16 | 27 | **25** | 25 | 3 | 4 | 12 | neg |
| PSA-162 | PSA | *SAMN57560091* | NRC | negativ | *bla*_OXA-486_, *bla*_PDC-3_ | ≥ 16 | ≥ 16 | 26 | 27 | 26 | 5 | 6 | 11 | neg |
| PSA-163 | PSA | *SAMN57560092* | NRC | negativ | *bla*_OXA-847_, *bla*_PDC-24_ | ≥ 16 | ≥ 16 | 25 | 28 | 27 | 3 | 4 | 13 | neg |
| PSA-164 | PSA | *SAMN57560093* | NRC | negativ | *bla*_OXA-1245_, *bla*_PDC-1_ | ≥ 16 | ≥ 16 | 28 | 26 | 28 | 2 | 3 | 10 | neg |
| PSA-165 | PSA | *SAMN57560094* | NRC | negativ | *bla*_OXA-486_, *bla*_PDC-3_ | ≥ 16 | ≥ 16 | 27 | 26 | 26 | 2 | 3 | 13 | neg |
| PSA-166 | PSA | *SAMN57560095* | NRC | negativ | *bla*_OXA-913_, *bla*_PDC-37_ | ≥ 16 | ≥ 16 | 27 | 28 | 25 | 7 FP | 8 FP | 12 | neg |
| PSA-167 | PSA | *SAMN57560096* | NRC | negativ | *bla*_OXA-847_, *bla*_PDC-1_ | ≥ 16 | ≥ 16 | 27 | 28 | 26 | 2 | 3 | 12 | neg |
| PSA-168 | PSA | *SAMN57560097* | NRC | negativ | *bla*_OXA-50_, *bla*_PDC-3_ | ≥ 16 | ≥ 16 | 27 | 26 | 26 | 5 | 6 | 12 | neg |
| PSA-169 | PSA | *SAMN57560098* | NRC | negativ |  | NA | ≥ 16 | 28 | 28 | 26 | 5 | 6 | 10 | neg |
| PSA-170 | PSA | *SAMN57560099* | NRC | negativ | *bla*_OXA-904_, *bla*_PDC-97_ | ≥ 16 | 8 | 26 | **23** | 23 | 6 | 7 FP | 7 | neg |
| PSA-171 | PSA | *SAMN57560100* | NRC | negativ | *bla*_OXA-50_, *bla*_PDC_ | ≥ 16 | ≥ 16 | 29 | 30 | 28 | 3 | 4 | 5 FP | neg |
| PSA-172 | PSA | *SAMN57560101* | NRC | negativ | *bla*_OXA-486_, *bla*_PDC-24_ | ≥ 16 | 0,25 | 27 | 6 FP | 27 | 7 FP | 8 FP | 10 | neg |
| PSA-173 | PSA | *SAMN57560102* | NRC | negativ | *bla*_OXA-1035_, *bla*_PDC-19a_ | ≥ 16 | ≥ 16 | 27 | 30 | 25 | 4 | 5 | 13 | neg |
| PSA-174 | PSA | *SAMN57560103* | NRC | negativ | *bla*_OXA-1035_, *bla*_PDV-19a_ | ≥ 16 | ≥ 16 | 28 | 28 | 28 | 2 | 3 | 8 | neg |
| PSA-175 | PSA | *SAMN57560104* | NRC | negativ | *bla*_OXA-1028_, *bla*_PDC-3_ | ≥ 16 | ≥ 16 | 28 | 29 | 26 | 3 | 2 | 10 | neg |
| PSA-176 | PSA | *SAMN57560105* | Center 1 | *bla*_FIM-1_ | *bla*_CARB-2_, *bla*_OXA-205_, *bla*_OXA-488_, *bla*_PDC-35_ | ≥ 16 | ≥ 16 | 6 | 6 | 9 | 11 | 11 | 0 | pos |
| PSA-177 | PSA | *SAMN57560106* | Center 1 | negativ | *bla*_PAO99_, *bla*_OXA100_ | ≥ 16 | ≥ 16 | 6 FP | **23** | 6 FP | 5 | 6 | 8 | neg |
| PSA-178 | PSA | *SAMN57560107* | Center 1 | negativ | *bla*_PAO_, *bla*_OXA_ 494 | ≥ 16 | ≥ 16 | 25 | **25** | 25 | 5 | 5 | 11 | neg |
| PSA-179 | PSA | *SAMN57560108* | Center 1 | *bla*_GIM-1_ | *bla*_OXA99_, *bla*_PAO99_ | ≥ 16 | ≥ 16 | 6 | 6 | 6 | 14 | 15 | 5 | neg FN |
| PSA-180 | PSA | *SAMN57560109* | Center 1 | negativ | *bla*_PAO_, *bla*_OXA-50_ | 4 | ≥ 16 | 28 | 28 | 25 | 6 | 6 | 7 | neg |
| PSA-181 | PSA | *SAMN57560110* | Center 1 | *bla*_GIM-1_ | *bla*_OXA99_, *bla*_PAO_ | ≥ 16 | ≥ 16 | 6 | 6 | 6 | 10 | 12 | 0 | **neg** |
| PSA-182 | PSA | *SAMN57560111* | Center 1 | negativ | *bla*_OXA-395_, *bla*_PAO_ | ≥ 16 | ≥ 16 | 27 | 26 | 26 | 5 | 6 | 15 | neg |
| PSA-183 | PSA | *SAMN57560112* | Center 1 | negativ | *bla*_OXA-395_, *bla*_PAO_ | ≥ 16 | ≥ 16 | 26 | 27 | 28 | 4 | 5 | 11 | neg |
| PSA-184 | PSA | *SAMN57560113* | Center 1 | negativ | *bla*_OXA-50_, *bla*_PAO_ | 8 | ≥ 16 | 25 | 30 | 28 | 7 FP | 7 FP | 2 FP | neg |
| PSA-185 | PSA | *SAMN57560114* | Center 1 | negativ | *bla*_OXA-486_, *bla*_PAO_ | ≥ 16 | ≥ 16 | 26 | 29 | 27 | 3 | 3 | 10 | neg |
| PSA-186 | PSA | *SAMN57560115* | Center 1 | negativ | *bla*_PAO_, *bla*_OXA-488_, *bla*_OXA-2_ | ≥ 16 | ≥ 16 | 15 | 27 | 6 FP | 4 | 4 | 9 | neg |
| PSA-187 | PSA | *SAMN57560116* | Center 1 | negativ | *bla*_OXA-395_, *bla*_PAO_ | ≥ 16 | ≥ 16 | 35 | 31 | 29 | 2 | 3 | 6 | neg |
| PSA-188 | PSA | *SAMN57560117* | Center 1 | negativ | *bla*_PAO_, *bla*_OXA-395_ | 8 | ≥ 16 | 35 | 6 | 30 | 3 | 3 | 6 | neg |
| PSA-189 | PSA | *SAMN57560118* | Center 1 | negativ | *bla*_OXA-50_, *bla*_PAO_ | ≥ 16 | ≥ 16 | 30 | 33 | 29 | 2 | 2 | 5 FP | neg |
| PSA-190 | PSA | *SAMN57560119* | Center 1 | negativ | *bla*_OXA-395_, *bla*_PDC-30_ | ≥ 16 | ≥ 16 | 28 | 29 | 29 | 5 | 5 | 11 | neg |
| PSA-191 | PSA | *SAMN57560120* | Center 1 | negativ | *bla*_OXA-10_, *bla*_OXA-50_, *bla*_PAO_ | 8 | ≥ 16 | 23 | 29 | 23 | 2 | 4 | 1 FP | neg |
| PSA-192 | PSA | *SAMN57560121* | Center 1 | negativ | *bla*_OXA-395_, *bla*_PAO_ | ≥ 16 | ≥ 16 | 27 | 26 | 27 | 4 | 5 | 10 | neg |
| PSA-193 | PSA | *SAMN57560122* | Center 1 | negativ | *bla*_PAO_, *bla*_OXA-395_ | ≥ 16 | 8 | 28 | 30 | 26 | 1 | 2 | 13 | neg |
| PSA-194 | PSA | *SAMN57560123* | Center 1 | negativ | *bla*_OXA-488_, *bla*_PAO_ | ≥ 16 | ≥ 16 | 26 | 29 | 28 | 4 | 3 | 11 | neg |
| PSA-195 | PSA | *SAMN57560124* | Center 1 | negativ | *bla*_OXA-395_, *bla*_PAO_ | ≥ 16 | ≥ 16 | 29 | 27 | 25 | 2 | 3 | 12 | neg |
| PSA-196 | PSA | *SAMN57560125* | Center 1 | negativ | *bla*_OXA-50_, *bla*_PAO_ | ≥ 16 | ≥ 16 | 26 | 27 | 27 | 4 | 4 | 10 | neg |
| PSA-197 | PSA | *SAMN57560126* | Center 1 | *bla*_VIM-2_ | *bla*_PAO_, *bla*_OXA-100_, *bla*_VEB_ | ≥ 16 | ≥ 16 | 6 | 6 | 6 | 9 | 10 | 0 | pos |
| PSA-198 | PSA | *SAMN57560127* | Center 1 | negativ | *bla*_OXA-494_, *bla*_OXA-396_ | ≥ 16 | 8 | 27 | 28 | 29 | 4 | 4 | 10 | neg |
| PSA-199 | PSA | *SAMN57560128* | Center 1 | negativ | *bla*_OXA-50_, *bla*_PAO_ | ≥ 16 | ≥ 16 | 27 | 27 | 28 | 5 | 5 | 9 | neg |
| PSA-200 | PSA | *SAMN57560129* | Center 1 | negativ | *bla*_OXA-35_, *bla*_PAO_, *bla*_OXA-488_ | ≥ 16 | ≥ 16 | 6 FP | **24** | 9 FP | 1 | 1 | 3 FP | neg |
| PSA-201 | PSA | *SAMN57560130* | Center 1 | *bla*_VIM-2_ | *bla*_PAO_, *bla*_OXA-395_ | ≥ 16 | 4 | 6 | 6 | 6 | 17 | 16 | 2 | **neg** |
| PSA-202 | PSA | *SAMN57560131* | Center 1 | *bla*_VIM-2_ | *bla*_OXA-395_, *bla*_PAO_ | ≥ 16 | ≥ 16 | 6 | 6 | 6 | 9 | 10 | 0 | **neg** |
| PSA-203 | PSA | *SAMN57560132* | Center 1 | *bla*_VIM-2_ | *bla*_PAO_, *bla*_OXA-395_ | ≥ 16 | 4 | 6 | 6 | 6 | 19 | 19 | 3 | **neg** |
| PSA-204 | PSA | *SAMN57560133* | Center 1 | *bla*_VIM-2_ | *bla*_OXA-395_, *bla*_PAO_ | ≥ 16 | ≥ 16 | 6 | 17 | 6 | 9 | 10 | 0 | pos |
| PSA-205 | PSA | *SAMN57560134* | Center 1 | *bla*_NDM-1_ | *bla*_PAO_, *bla*_OXA-488_ | ≥ 16 | ≥ 16 | 6 | 6 | 6 | 17 | 17 | 0 | pos |
| PSA-206 | PSA | *SAMN57560135* | Center 1 | negativ | *bla*_PAO_, *bla*_OXA-395_ | ≥ 16 | ≥ 16 | 31 | 26 | 27 | 3 | 4 | 11 | neg |
| PSA-207 | PSA | *SAMN57560136* | Center 1 | negativ | *bla*_PAO_, *bla*_OXA-395_ | ≥ 16 | ≥ 16 | 31 | 35 | 29 | 3 | 4 | 6 | neg |
| PSA-208 | PSA | *SAMN57560137* | Center 1 | negativ | *bla*_OXA-488_ | ≥ 16 | ≥ 16 | 29 | 27 | 28 | 5 | 5 | 11 | neg |
| PSA-209 | PSA | *SAMN57560138* | Center 1 | negativ | *bla*_PAO_, *bla*_OXA-395_ | ≥ 16 | ≥ 16 | 28 | 27 | 25 | 5 | 6 | 10 | neg |
| PSA-210 | PSA | *SAMN57560139* | Center 1 | negativ | *bla*_PAO_, *bla*_OXA-486_ | ≥ 16 | ≥ 16 | 30 | 27 | 27 | 1 | 2 | 10 | neg |
| PSA-211 | PSA | *SAMN57560140* | Center 1 | negativ | *bla*_PAO_, *bla*_OXA-494_, *bla*_OXA-396_ |  |  | 28 | 29 | 25 | 3 | 4 | 8 | neg |
| PSA-212 | PSA | *SAMN57560141* | Center 1 | *bla*_VIM-2_ | *bla*_PAO_, *bla*_OXA-395_, | ≥ 16 | 4 | 6 | 6 | 6 | 18 | 17 | 1 | pos |
| PSA-213 | PSA | *SAMN57560142* | Center 1 | negativ | *bla*_PAO_, *bla*_OXA-395_ | ≥ 16 | ≥ 16 | 28 | 28 | 27 | 7 FP | 9 FP | 12 | neg |
| PSA-214 | PSA | *SAMN57560143* | Center 1 | negativ | *bla*_OXA-50_, *bla*_PAO_ | ≥ 16 | ≥ 16 | 32 | 31 | 28 | 5 | 5 | 10 | neg |
| PSA-215 | PSA | *SAMN57560144* | Center 1 | negativ | *bla*_PAO_, *bla*_OXA-50_ | 8 | ≥ 16 | 27 | 32 | 31 | 6 | 5 | 0 FP | neg |
| PSA-216 | PSA | *SAMN57560145* | Center 1 | *bla*_VIM-2_ | *bla*_PAO_, *bla*_OXA-395_ | ≥ 16 | ≥ 16 | 6 | 6 | 19 FN | 10 | 12 | 0 | pos |
| PSA-217 | PSA | *SAMN57560146* | Center 1 | negativ | *bla*_OXA-50_, *bla*_PAO_ | ≥ 16 | ≥ 16 | 29 | 26 | 29 | 7 FP | 9 FP | 11 | neg |
| PSA-218 | PSA | *SAMN57560147* | Center 1 | negativ | *bla*_OXA-486_, *bla*_PAO_ | ≥ 16 | ≥ 16 | 28 | 29 | 30 | 4 | 5 | 9 | neg |
| PSA-219 | PSA | *SAMN57560148* | Center 1 | negativ | *bla*_OXA-50_, *bla*_PAO_ | ≥ 16 | ≥ 16 | 30 | 27 | 26 | 4 | 5 | 11 | neg |
| PSA-220 | PSA | *SAMN57560149* | Center 1 | negativ | *bla*_OXA-395_, *bla*_PAO_ | ≥ 16 | ≥ 16 | 28 | 30 | 28 | 4 | 4 | 8 | neg |
| PSA-221 | PSA | *SAMN57560150* | Center 1 | negativ | *bla*_OXA-486_, *bla*_PAO_ | ≥ 16 | ≥ 16 | 29 | 26 | 27 | 4 | 5 | 9 | neg |
| PSA-222 | PSA | *SAMN57560151* | Center 1 | negativ | *bla*_OXA-486_, *bla*_PAO_ | ≥ 16 | ≥ 16 | 26 | 27 | 30 | 4 | 4 | 9 | neg |
| PSA-223 | PSA | *SAMN57560152* | Center 1 | negativ | *bla*_OXA-395_, *bla*_PAO_ | ≥ 16 | ≥ 16 | 26 | 27 | 28 | 4 | 3 | 11 | neg |
| PSA-224 | PSA | *SAMN57560153* | Center 1 | negativ | *bla*_OXA-396_, *bla*_PAO_ | ≥ 16 | ≥ 16 | 29 | 28 | 24 | 6 | 7 FP | 7 | neg |
| PSA-225 | PSA | *SAMN57560154* | Center 1 | negativ | *bla*_OXA-50_, *bla*_PAO_ | ≥ 16 | ≥ 16 | 28 | 27 | 26 | 4 | 5 | 10 | neg |
| PSA-226 | PSA | *SAMN57560155* | Center 1 | negativ | *bla*_OXA-488_, *bla*_PAO_ | ≥ 16 | ≥ 16 | 27 | 30 | 29 | 4 | 4 | 8 | neg |
| PSA-227 | PSA | *SAMN57560156* | Center 1 | negativ | *bla*_OXA-488_, *bla*_PAO_ | ≥ 16 | ≥ 16 | 27 | 29 | 28 | 6 | 7 FP | 10 | neg |
| PSA-228 | PSA | *SAMN57560157* | Center 1 | negativ | *bla*_OXA-488_, *bla*_PAO_ | ≥ 16 | ≥ 16 | 28 | **25** | 27 | 4 | 4 | 10 | neg |
| PSA-229 | PSA | *SAMN57560158* | Center 1 | negativ | *bla*_PAO_, *bla*_OXA-50_, *bla*_VEB-1_ | ≥ 16 | ≥ 16 | 27 | 6 FP | 28 | 6 | 6 | 12 | neg |
| PSA-230 | PSA | *SAMN57560159* | Center 1 | negativ | *bla*_OXA-395_, *bla*_PAO_ | ≥ 16 | ≥ 16 | 6 FP | 6 FP | 6 FP | 11 FP | 11 FP | 0 FP | **neg** |
| PSA-231 | PSA | *SAMN57560160* | Center 1 | *bla*_NDM-1_ | *bla*_OXA-395_, *bla*_PAO_ | ≥ 16 | ≥ 16 | 6 | 6 | 6 | 11 | 12 | 0 | pos |
| PSA-232 | PSA | *SAMN57560161* | Center 1 | negativ | *bla*_OXA-50_, *bla*_PAO_ | ≥ 16 | ≥ 16 | 26 | 6 FP | 25 | 6 | 6 | 13 | neg |
| PSA-233 | PSA | *SAMN57560162* | Center 1 | negativ | *bla*_PAO_, *bla*_OXA-494_, *bla*_OXA-396_ | ≥ 16 | ≥ 16 | 28 | 27 | 28 | 4 | 4 | 8 | neg |
| PSA-234 | PSA | *SAMN57560163* | Center 1 | *bla*_VIM-1_ | *bla*_PAO_, *bla*_OXA-50_ | ≥ 16 | ≥ 16 | 6 | 6 | 6 | 8 | 9 | 0 | neg |
| PSA-235 | PSA | *SAMN57560164* | Center 1 | *bla*_VIM-4_, *bla*_NDM-1_ | *bla*_PAO_, *bla*_OXA-395_ | ≥ 16 | ≥ 16 | 6 | 6 | 6 | 11 | 11 | 0 | pos |
| PSA-236 | PSA | *SAMN57560165* | Center 1 | negativ | *bla*_OXA-494_, *bla*_OXA-396_ | ≥ 16 | ≥ 16 | 32 | 32 | 26 | 2 | 3 | 10 | neg |
| PSA-237 | PSA | *SAMN57560166* | Center 1 | *bla*_VIM-2_ | *bla*_PAO_, *bla*_OXA-395_, *bla* OXA-10 | ≥ 16 | ≥ 16 | 15 | 14 | 7 | 9 | 10 | 0 | pos |
| PSA-238 | PSA | *SAMN57560167* | Center 1 | negativ | *bla*_Pao_, *bla*_OXA-50_ | ≥ 16 | ≥ 16 | 26 | 29 | 28 | 5 | 5 | 10 | neg |
| PSA-239 | PSA | *SAMN57560168* | Center 1 | negativ | *bla*_PAO_, *bla*_OXA-395_ | ≥ 16 | ≥ 16 | 6 FP | 6 FP | 24 | 4 | 4 | 13 | neg |
| PSA-240 | PSA | *SAMN57560169* | Center 1 | negativ | *bla*_PAO_, *bla*_OXA-395_ | ≥ 16 | ≥ 16 | 24 | 6 FP | 24 | 5 | 6 | 10 | neg |
| PSA-241 | PSA | *SAMN57560170* | Center 1 | negativ | *bla*_PAO_, *bla*_OXA-488_ | ≥ 16 | ≥ 16 | 29 | 28 | 27 | 2 | 4 | 6 | neg |
| PSA-242 | PSA | *SAMN57560171* | Center 1 | negativ | *bla*_PAO_, *bla*_OXA-50_, *bla*_VEB-1_ | ≥ 16 | ≥ 16 | 29 | 28 | 31 | 6 | 7 FP | 14 | neg |
| PSA-243 | PSA | *SAMN57560172* | Center 1 | *bla*_NDM-1_ | *bla*_PAO_, *bla*_OXA-395_ | ≥ 16 | ≥ 16 | 6 | 6 | 6 | 10 | 12 | 0 | pos |
| PSA-244 | PSA | *SAMN57560173* | Center 1 | *bla*_NDM-1_ | *bla*_PAO_, *bla*_OXA-395_ | ≥ 16 | ≥ 16 | 6 | 20 | 6 | 9 | 11 | 0 | pos |
| PSA-245 | PSA | *SAMN57560174* | Center 1 | negativ | *bla*_PAO_, *bla*_OXA-486_ | ≥ 16 | ≥ 16 | 23 | **25** | 28 | 3 | 4 | 14 | neg |
| PSA-246 | PSA | *SAMN57560175* | Center 1 | negativ | *bla*_PAO_, *bla*_OXA-486_ | ≥ 16 | ≥ 16 | 27 | 26 | 30 | 2 | 2 | 8 | neg |
| PSA-247 | PSA | *SAMN57560176* | Center 1 | negativ | *bla*_PAO_, *bla*_OXA-486_ | ≥ 16 | ≥ 16 | 28 | 27 | 27 | 5 | 6 | 11 | neg |
| PSA-248 | PSA | *SAMN57560177* | Center 1 | negativ | *bla*_OXA-50_, *bla*_PAO_ | ≥ 16 | ≥ 16 | 28 | 28 | 28 | 3 | 4 | 11 | neg |
| PSA-249 | PSA | *SAMN57560178* | Center 2 | negativ | *bla*_OXA-17_, *bla*_OXA-488_, *bla*_PDC-35_ | 2 | 1 | 28 | 30 | 27 | 0 | 0 | 4 FP | neg |
| PSA-250 | PSA | *SAMN57560179* | Center 2 | negativ | *bla*_OXA-395_, *bla*_PDC-3_ | ≥ 16 | ≥ 16 | 25 | 27 | 28 | 7 FP | 8 FP | 10 | **neg** |
| PSA-251 | PSA | *SAMN57560180* | Center 2 | negativ | *bla*_OXA-486_, *bla*_PAO_ | ≥ 16 | ≥ 16 | 27 | 28 | 25 | 12 FP | 13 FP | 14 | neg |
| PSA-252 | PSA | *SAMN57560181* | Center 2 | negativ | *bla*_OXA-488_, *bla*_PAO_ | 4 | 3 | 26 | 28 | 27 | 8 FP | 7 FP | 6 | neg |
| PSA-253 | PSA | *SAMN57560182* | Center 2 | negativ | *bla*_PAO_, *bla*_OXA-494_, *bla*_OXA-396_ | ≥ 16 | NA | 26 | 29 | 26 | 9 FP | 9 FP | 7 | neg |
| PSA-254 | PSA | *SAMN57560183* | Center 2 | negativ | *bla*_OXA-50_, *bla*_PAO_ | ≥ 16 | 4 | 26 | 30 | 23 | 0 | 1 | 6 | neg |
| PSA-255 | PSA | *SAMN57560184* | Center 2 | negativ | *bla*_PAO_, *bla*_OXA-486_ | 8 | 4 | 23 | **23** | 22 | 2 | 2 | 9 | neg |
| PSA-256 | PSA | *SAMN57560185* | Center 2 | negativ | *bla*_OXA-488_, *bla*_OXA-396_, *bla*_PAO_ | ≥ 16 | ≥ 16 | 28 | 30 | 24 | 5 | 6 | 9 | neg |
| PSA-257 | PSA | *SAMN57560186* | Center 2 | negativ | *bla*_PAO_, *bla*_OXA-50_ | 8 | ≥ 16 | 27 | 28 | 26 | 5 | 6 | 11 | neg |
| PSA-258 | PSA | *SAMN57560187* | Center 2 | negativ |  | ≥ 16 | 8 | 27 | 31 | 26 | 5 | 7 FP | 14 | neg |
| PSA-259 | PSA | *SAMN57560188* | Center 2 | negativ | *bla*_OXA-395_, *bla*_PAO_ | ≥ 16 | ≥ 16 | 25 | 31 | 25 | 7 FP | 8 FP | 13 | neg |
| PSA-260 | PSA | *SAMN57560189* | Center 2 | negativ | *bla*_OXA-494_, *bla*_PDC-8_ | 1 | 0,5 | 30 | 30 | 28 | 1 | 1 | 3 FP | neg |
| PSA-261 | PSA | *SAMN57560190* | Center 2 | negativ | *bla*_OXA-486_, *bla*_PDC-3_ | ≥ 16 | 4 | 25 | **24** | 24 | 6 | 6 | 14 | neg |
| PSA-262 | PSA | *SAMN57560191* | Center 2 | negativ | *bla*_OXA-395_, *bla*_PAO_ | 2 | ≤ 0,25 | 27 | 29 | 24 | 1 | 1 | 12 | neg |
| PSA-263 | PSA | *SAMN57560192* | Center 2 | negativ | *bla*_OXA-488_, *bla*_PAO_ | 1 | 1 | 26 | 29 | 25 | 1 | 0 | 1 FP | neg |
| PSA-264 | PSA | *SAMN57560193* | Center 2 | negativ | *bla*_OXA-396_ | ≥ 16 | ≥ 16 | 25 | 29 | 26 | 4 | 5 | 8 | neg |
| PSA-265 | PSA | *SAMN57560194* | Center 2 | negativ | *bla*_OXA-847_, *bla*_PDC-3_ | ≥ 16 | ≥ 16 | 29 | 29 | 29 | 7 FP | 7 FP | 15 | neg |
| PSA-266 | PSA | *SAMN57560195* | Center 2 | *bla*_VIM-2_ | *bla*_OXA-395_, *bla*_PDC-3_ | ≥ 16 | ≥ 16 | 6 | 6 | 6 | 12 | 12 | 0 | pos |
| PSA-267 | PSA | *SAMN57560196* | Center 2 | negativ | *bla*_OXA-488_, *bla*_PAO_ | ≥ 16 | ≥ 16 | 26 | 31 | 25 | 7 FP | 8 FP | 10 | neg |
| PSA-268 | PSA | *SAMN57560197* | Center 2 | negativ | *bla*_PAO_, *bla*_OXA-396_ | 8 | ≥ 16 | 28 | 28 | 28 | 2 | 2 | 3 FP | neg |
| PSA-269 | PSA | *SAMN57560198* | Center 2 | negativ | *bla*_OXA-50_, *bla*_PDC-31_ | ≥ 16 | ≥ 16 | 28 | 26 | 29 | 4 | 5 | 10 | neg |
| PSA-270 | PSA | *SAMN57560199* | Center 2 | negativ | *bla*_OXA-486_, *bla*_PDC-31_ | 2 | 4 | 26 | **24** | 27 | 5 | 4 | 8 | neg |
| PSA-271 | PSA | *SAMN57560200* | Center 2 | negativ | *bla*_OXA-488_, *bla*_PAO_ | ≥ 16 | ≥ 16 | 25 | 27 | 28 | 1 | 2 | 8 | neg |
| PSA-272 | PSA | *SAMN57560201* | Center 2 | negativ | *bla*_PAO_, *bla*_OXA-396_, *bla*_OXA-494_ | 8 | 2 | 29 | 28 | 28 | 2 | 1 | 3 FP | neg |
| PSA-273 | PSA | *SAMN57560202* | Center 2 | negativ | *bla*_PAO_, *bla*_OXA-494_, *bla*_OXA-396_ | 1 | ≥ 16 | 25 | **23** | 26 | 3 | -1 | 6 | neg |
| PSA-274 | PSA | *SAMN57560203* | Center 2 | negativ |  | 2 | 2 | 26 | 27 | 26 | 1 | 0 | 3 FP | neg |
| PSA-275 | PSA | *SAMN57560204* | Center 2 | negativ | *bla*_OXA-395_, *bla*_PAO_ | 2 | 1 | 26 | 27 | 26 | 3 | 2 | 2 FP | neg |
| PSA-276 | PSA | *SAMN57560205* | Center 2 | negativ | *bla*_PAO_, *bla*_OXA-488_ | ≥ 16 | ≥ 16 | 26 | 28 | 28 | 6 | 6 | 2 FP | neg |
| PSA-277 | PSA | *SAMN57560206* | Center 2 | negativ | *bla*_OXA-1028_, *bla*_PDC-3_ | 8 | ≥ 16 | 28 | 28 | 30 | 4 | 5 | 12 | neg |
| PSA-278 | PSA | *SAMN57560207* | Center 2 | negativ | *bla*_PAO_, *bla*_OXA-486_ | ≥ 16 | ≥ 16 | 25 | 28 | 28 | 4 | 6 | 10 | neg |
| PSA-279 | PSA | *SAMN57560208* | Center 2 | negativ | *bla*_OXA-494_, *bla*_PDC_ | ≥ 16 | 4 | 25 | 28 | 25 | 2 | 2 | 13 | neg |
| PSA-280 | PSA | *SAMN57560209* | Center 2 | negativ | *bla*_PAO_, *bla*_OXA-494_, *bla*_OXA-396_ | 8 | 8 | 28 | 26 | 34 | 5 | 7 FP | 8 | neg |
| PSA-281 | PSA | *SAMN57560210* | Center 2 | negativ | *bla*_OXA-486_, *bla*_PDC-3_ | 8 | 4 | 27 | **24** | 26 | 3 | 5 | 10 | neg |
| PSA-282 | PSA | *SAMN57560211* | Center 2 | *bla*_VIM-2_ | *bla*_PAO_, *bla*_OXA-395_ | ≥ 16 | ≥ 16 | 6 | 6 | 6 | 11 | 13 | 0 | pos |
| PSA-283 | PSA | *SAMN57560212* | Center 2 | negativ | *bla*_OXA-486_, *bla*_PDV-24_ | ≥ 16 | ≥ 16 | 27 | 29 | 25 | 5 | 6 | 10 | neg |
| PSA-284 | PSA | *SAMN57560213* | Center 2 | negativ | *bla*_OXA-50_, *bla*_PDC-103_ | 8 | ≥ 16 | 29 | 28 | 29 | 5 | 6 | 11 | neg |
| PSA-285 | PSA | *SAMN57560214* | Center 2 | *bla*_VIM-2_ | *bla*_PAO_, *bla*_OXA-395_ | ≥ 16 | ≥ 16 | 6 | 6 | 6 | 12 | 12 | 0 | pos |
| PSA-286 | PSA | *SAMN57560215* | Center 2 | negativ | *bla*_OXA-847_, *bla*_PDC-3_ | 2 | 2 | 28 | 29 | 30 | 2 | 2 | 5 FP | neg |
| PSA-287 | PSA | *SAMN57560216* | Center 2 | negativ | *bla*_OXA-846_, *bla*_PDC-56_ | ≥ 16 | 8 | 28 | **25** | 30 | 8 FP | 8 FP | 15 | neg |
| PSA-288 | PSA | *SAMN57560217* | Center 2 | negativ | *bla*_OXA-395_, *bla*_PDC-5_ | ≥ 16 | 8 | 22 | 29 | 22 | 4 | 4 | 13 | neg |
| PSA-289 | PSA | *SAMN57560218* | Center 2 | negativ | *bla*_OXA-913_, *bla*_PDC-37_ | ≥ 16 | 8 | 29 | 28 | 28 | 2 | 3 | 12 | neg |
| PSA-290 | PSA | *SAMN57560219* | Center 2 | negativ | *bla*_OXA-486_, *bla*_PAO_ | ≥ 16 | ≥ 16 | 27 | 30 | 25 | 4 | 6 | 9 | neg |
| PSA-291 | PSA | *SAMN57560220* | Center 2 | negativ | *bla*_OXA-50_, *bla*_PAO_ | ≥ 16 | ≥ 16 | 27 | 28 | 27 | 4 | 6 | 12 | neg |
| PSA-292 | PSA | *SAMN57560221* | Center 2 | negativ | *bla*_OXA-486_, *bla*_PAO_ | ≥ 16 | ≥ 16 | 26 | 29 | 25 | 6 | 7 FP | 11 | neg |
| PSA-293 | PSA | *SAMN57560222* | Center 2 | negativ | *bla*_OXA-50_, *bla*_PAO_ | 8 | ≥ 16 | 28 | 29 | 27 | 6 | 7 FP | 7 | neg |
| PSA-294 | PSA | *SAMN57560223* | Center 2 | negativ | *bla*_PAO_, *bla*_OXA-50_ | ≥ 16 | ≥ 16 | 27 | 30 | 28 | 7 | 9 FP | 13 | neg |
| PSA-295 | PSA | *SAMN57560224* | Center 2 | negativ | *bla*_OXA-396_, *bla*_PAO_ | ≥ 16 | 8 | 26 | 28 | 24 | 10 FP | 11 FP | 10 | neg |
| PSA-296 | PSA | *SAMN57560225* | Center 2 | negativ | *bla*_PAO_, *bla*_OXA-486_ | 8 | ≥ 16 | 16 | 6 FP | 21 | 3 | 4 | 10 | neg |
| PSA-297 | PSA | *SAMN57560226* | Center 2 | negativ | *bla*_OXA-396_, *bla*_PAO_ | 8 | ≥ 16 | 26 | 29 | 28 | 6 | 7 FP | 11 | neg |
| PSA-298 | PSA | *SAMN57560227* | Center 2 | negativ | *bla*_OXA-488_, *bla*_PAO_ | 1 | 4 | 27 | 29 | 26 | 2 | 3 | 0 FP | neg |
| PSA-299 | PSA | *SAMN57560228* | Center 2 | negativ | *bla*_OXA-488_, *bla*_PAO_ | 2 | ≤ 0,25 | 26 | 30 | 32 | 3 | 3 | 4 FP | **neg** |
| PSA-300 | PSA | *SAMN57560229* | Center 2 | negativ | *bla*_PAO_, *bla*_OXA-395_ | ≥ 16 | ≥ 16 | 29 | 30 | 27 | 7 FP | 7 FP | 11 | neg |
| PSA-301 | PSA | *SAMN57560230* | Center 2 | negativ | *bla*_OXA-494_, *bla*_OXA-396_ | ≥ 16 | ≥ 16 | 25 | 26 | 25 | 7 FP | 8 FP | 14 | neg |
| PSA-302 | PSA | *SAMN57560231* | Center 2 | negativ | *bla*_OXA-50_, *bla*_OXA-494_, *bla*_OXA-396_, *bla*_PAO_ | ≥ 16 | 2 | 23 | 28 | 23 | 9 FP | 9 FP | 10 | neg |
| PSA-303 | PSA | *SAMN57560232* | Center 2 | negativ | *bla*_PAO_ | 8 | ≥ 16 | 27 | 29 | 26 | 4 | 5 | 8 | neg |
| PSA-304 | PSA | *SAMN57560233* | Center 2 | negativ | *bla*_OXA-396_, *bla*_PAO_ | ≥ 16 | ≥ 16 | 28 | 27 | 28 | 5 | 6 | 12 | **neg** |
| PSA-305 | PSA | *SAMN57560234* | Center 2 | negativ | *bla*_PAO_, *bla*_OXA-494_, *bla*_OXA-396_ | ≥ 16 | ≥ 16 | 26 | 27 | 27 | 3 | 4 | 9 | neg |
| PSA-306 | PSA | *SAMN57560235* | Center 2 | *bla*_VIM-2_ | *bla*_PAO_, *bla*_OXA-395_ | ≥ 16 | ≥ 16 | 6 | 6 | 6 | 11 | 13 | 0 | pos |
| PSA-307 | PSA | *SAMN57560236* | Center 2 | negativ | *bla*_OXA-488_, *bla*_PAO_ | 2 | 2 | 25 | 30 | 24 | 1 | 2 | 2 FP | neg |
| PSA-308 | PSA | *SAMN57560237* | Center 2 | negativ | *bla*_PAO_, *bla*_OXA-494_, *bla*_OXA-396_ | 1 | 1 | 26 | 26 | 26 | 5 | 6 | 5 FP | neg |
| PSA-309 | PSA | *SAMN57560238* | Center 2 | negativ | *bla*_OXA-488_, *bla*_PAO_ | ≥ 16 | ≥ 16 | 26 | 27 | 27 | 5 | 7 FP | 5 FP | neg |
| PSA-310 | PSA | *SAMN57560239* | Center 2 | negativ | *bla*_PAO_, *bla*_OXA-396_ | 8 | NA | 27 | 32 | 26 | 3 | 4 | 8 | neg |
| PSA-311 | PSA | *SAMN57560240* | Center 2 | negativ | *bla*_OXA-50_, *bla*_PAO_ | ≥ 16 | ≥ 16 | 27 | **25** | 24 | 0 | 0 | 11 | neg |
| PSA-312 | PSA | *SAMN57560241* | Center 2 | negativ | *bla*_OXA-488_ | ≥ 16 | 8 | 27 | 31 | 30 | 5 | 5 | 13 | **neg** |
| PSA-313 | PSA | *SAMN57560242* | Center 2 | negativ | *bla*_PAO_, *bla*_OXA-395_ | 1 | 1 | 28 | 30 | 28 | 2 | 3 | 4 FP | neg |
| PSA-314 | PSA | *SAMN57560243* | Center 2 | negativ | *bla*_OXA-486_, *bla*_PAO_ | ≥ 16 | ≥ 16 | 28 | 28 | 28 | 4 | 6 | 12 | neg |
| PSA-315 | PSA | *SAMN57560244* | Center 2 | negativ | *bla*_PAO_, *bla*_OXA-50_ | NA | NA | 27 | 30 | 27 | 2 | 6 | 5 FP | neg |
| PSA-316 | PSA | *SAMN57560245* | Center 2 | negativ | *bla*_OXA-488_, *bla*_PAO_ | ≥ 16 | ≥ 16 | 28 | 27 | 28 | 9 FP | 9 FP | 14 | neg |
| PSA-317 | PSA | *SAMN57560246* | Center 2 | negativ | *bla*_OXA-50_, *bla*_PAO_ | ≥ 16 | ≥ 16 | 27 | 29 | 26 | 4 | 6 | 10 | neg |
| PSA-318 | PSA | n.d. | Center 1 | *bla*_GIM-1_ |  | ≥ 16 | ≥ 16 | 6 | 6 | 6 | 10 | 12 | 0 | pos |
| PSA-319 | PSA | n.d. | NRC | *bla*_VIM-1_ |  | ≥ 16 | ≥ 16 | 6 | 6 | 6 | 9 | 10 | 0 | neg FN |
| PSA-320 | PSA | n.d. | NRC | negativ |  | 8 | ≥ 16 | 23 | 30 | 22 | 4 | 5 | 9 | neg |

Characterization of carbapenemase-producing isolates and evaluation of β-lactam susceptibility. Minimal inhibitory concentrations were determined by VITEK 2. n.d. = not determined. Results for mCIM, sCIM, and mzCIM represent measurements of the inhibition zone diameter (IZD, mm). Δd EDTA 750 µg and Δd EDTA 930 µg represent the differences in mm between imipenem inhibition zones with and without EDTA. Δd IC-4000 represents the difference in imipenem inhibition zones with and without cloxacillin. Carba NP results are expressed as positive or negative for carbapenemase production. Indeterminate results are underlined and bold. Abbreviations: PSA = *Pseudomonas aeruginosa*; NRC = National Reference Center for Gram-negative hospital pathogens Germany; IMP = imipenem; MEM = meropenem; Δd = difference in inhibition zone diameter; mCIM = Modified Carbapenem Inactivation Method; sCIM = Simplified Carbapenem Inactivation Method; mzCIM = Modified Zinc Supplemented Carbapenem Inactivation Method; EDTA = ethylenediaminetetraacetic acid; FP = false positive result; FN = false negative result.
